# Adaptive Balance in Posterior Cerebellum

**DOI:** 10.3389/fneur.2021.635259

**Published:** 2021-03-09

**Authors:** Neal H. Barmack, Vito Enrico Pettorossi

**Affiliations:** ^1^Department of Physiology & Pharmacology, Oregon Health & Science University, Portland, OR, United States; ^2^Section of Human Physiology and Biochemistry, Department of Experimental Medicine, University of Perugia, Perugia, Italy

**Keywords:** vestibular, inferior olive, Purkinje cell, microRNA, semicircular canal, otolith, corticotropin releasing factor, cerebellum

## Abstract

Vestibular and optokinetic space is represented in three-dimensions in vermal lobules IX-X (uvula, nodulus) and hemisphere lobule X (flocculus) of the cerebellum. Vermal lobules IX-X encodes gravity and head movement using the utricular otolith and the two vertical semicircular canals. Hemispheric lobule X encodes self-motion using optokinetic feedback about the three axes of the semicircular canals. Vestibular and visual adaptation of this circuitry is needed to maintain balance during perturbations of self-induced motion. Vestibular and optokinetic (self-motion detection) stimulation is encoded by cerebellar climbing and mossy fibers. These two afferent pathways excite the discharge of Purkinje cells directly. Climbing fibers preferentially decrease the discharge of Purkinje cells by exciting stellate cell inhibitory interneurons. We describe instances adaptive balance at a behavioral level in which prolonged vestibular or optokinetic stimulation evokes reflexive eye movements that persist when the stimulation that initially evoked them stops. Adaptation to prolonged optokinetic stimulation also can be detected at cellular and subcellular levels. The transcription and expression of a neuropeptide, corticotropin releasing factor (CRF), is influenced by optokinetically-evoked olivary discharge and may contribute to optokinetic adaptation. The transcription and expression of microRNAs in floccular Purkinje cells evoked by long-term optokinetic stimulation may provide one of the subcellular mechanisms by which the membrane insertion of the GABAA receptors is regulated. The neurosteroids, estradiol (E2) and dihydrotestosterone (DHT), influence adaptation of vestibular nuclear neurons to electrically-induced potentiation and depression. In each section of this review, we discuss how adaptive changes in the vestibular and optokinetic subsystems of lobule X, inferior olivary nuclei and vestibular nuclei may contribute to the control of balance.

## Introduction

Vestibular and optokinetic space is represented in three-dimensions in vermal lobules IX-X (uvula, nodulus) and hemispheric lobule X (flocculus) of the cerebellum. The coordinates of these spaces correspond physically to the planar orientation of the three semicircular canals and to the planes of action of the three pairs of extraocular muscles (1–5). Although reflexive and centrally-initiated movements can be made without a fully functional cerebellum, its circuitry maintains a central representation of head position in space and is capable of adapting to modifications in vestibular and optokinetic stimulation.

In this review we describe the anatomy, physiology and certain molecular features of the vestibular and optokinetic afferent pathways to cerebellar Purkinje neurons. These pathways include information processing at the levels of the medial accessory optic system, vestibular nuclei, nuclei of the inferior olive and cellular features of the cerebellar cortical interneurons. At each step along the way we consider evidence that each nuclear structure may contribute to the adaptive balance of the vestibular system.

### Vestibular Primary Afferent Fibers Project to Ipsilateral Vestibular Nuclei and Vermal Lobules IX-X

**Figure 1:**
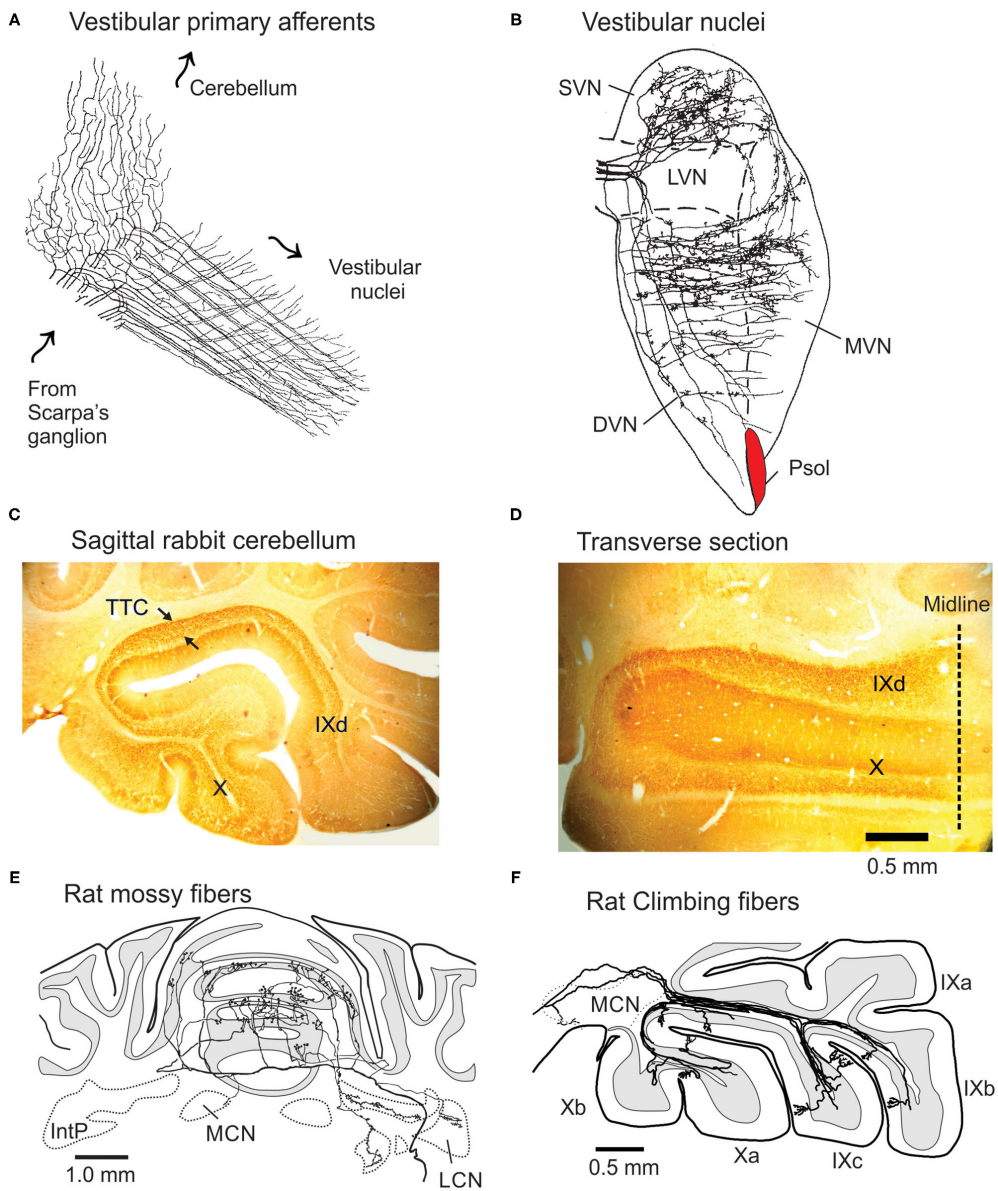
Projections of vestibular primary afferents to cerebellum and vestibular nuclei. **(A)** Vestibular primary afferents from Scarpa's ganglion terminate in the vestibular nuclei after thin collateral branches separate and project to the granule cell layer of lobules IX-X as MFTs. Arrows indicate direction of signal propagation. Modified from Cajal (6). **(B)** Horizontal section through the vestibular complex illustrates the terminal fields of five horizontal semicircular canal afferents, intra-axonally labeled with HRP in rat. Modified from Sato and Sasaki (7). **(C)** The C fragment of tetanus toxin (TTC) injected into the left labyrinth of rabbit, orthogradely labels MFTs in lobules IX-X. Arrows show the profuse labeling in sagittal section. Note absence of labeling in other folia. **(D)** In horizontal section TTC-labeled mossy fibers stay within the bounds of left lobules IX-X. Modified from Barmack et al. (8). **(E)** Transverse section of the projection pattern of a BDA-labeled lateral reticular nucleus neuron of the rat. It projects bilaterally as a profusely branching mossy fiber to the anterior cerebellar vermis. **(F)** Sagittal view of several BDA-labeled climbing fibers that project in narrow sagittal bands to the contralateral lobules IX-X. DVN, LVN, MVN, and SVN, descending, lateral, medial, and superior vestibular nuclei; IntP, Interpositus nucleus; LCN and MCN, lateral and medial cerebellar nucleus; Psol, parasolitary nucleus. Modified from Wu et al. (9).

Vestibular primary afferents sprout a thinner collateral branch as the primary afferent passes through the superior and lateral vestibular nuclei and terminates on granule cells in the ipsilateral vermal lobules IXd-X (Figures 1A,C,D). The main branch terminates in each of the five vestibular nuclei. These include the descending, lateral, medial and superior nuclei and the parasolitary nucleus (Psol) (10–14) (Figure 1B). The thinner collateral branch of the vestibular nerve branches again within the cerebellar cortex and distributes its terminals both sagittally and medio-laterally within vermal lobules IXd-X. Vestibular mossy fibers account for ~90% of the total mossy fiber projection to vermal lobules IXd-X (8, 15–23). The mossy fiber branching pattern is illustrated best by the spatial patterning of mossy fiber terminals (MFTs) that originate from the lateral reticular nucleus (LRN) after labeling with biotin dextran amine (BDA) (9) (Figure 1E). A single mossy fiber branch forms ~40 MFTs that contact one of a granule cell's 3-6 dendrites of as many as ~15 granule cells. These synapses include a descending Golgi cell axon terminal (6, 24). In total, a single mossy fiber makes synaptic contact with ~600 granule cells (25).

Each vestibular endorgan, the three semicircular canals, utricular and saccular otoliths, projects to a principal folium within vermal lobules IX-X. However, the projection of MFTs is not restricted to a single folium, but includes other folia within lobules IX-X and as well. MFTs from, say the left posterior semicircular canal (LPC), project primarily to left vermal lobule X, but also, more sparsely to left vermal lobule IXd. The left saccule projects to the left vermal lobule IX, but more sparsely to the left vermal lobule X (23). This pattern of distributed projections of vestibular primary afferents could account for the “patchy-mosaic” of granule cell receptive fields described for cutaneous cerebellar cortical areas that appear spatially discontinuous (26).

### Parallel Fibers Distribute Vestibular Primary Afferent Signals Medio-Laterally Within Vermal Folia

Granule cell axons ascend through the Purkinje cell layer and bifurcate into parallel fibers that run through the planar dendritic trees of Purkinje cells in the molecular layer. The lengths of parallel fibers, ~5 mm (rat) and ~7 mm (cat), adds to the dispersion of mossy fiber signals and assures that fractions of the parallel fibers that traverse dendrites of each Purkinje cell in lobules IXd-X convey information that originates from each of the ipsilateral vestibular endorgans (27–29). While parallel fibers are numerous, their signals are weak. Fewer than 10% of parallel fibers evoke a detectable synaptic response in Purkinje cells. Based on these measurements, it is estimated that the synchronous discharge of ~150 parallel fibers is necessary to evoke a Purkinje cell action potential (30).

A small number of granule cell axons ascend through the Purkinje cell layer and into the molecular layer and make synaptic contact with the overlying Purkinje cell dendritic tree prior to bifurcating into parallel fibers. These ascending axons could potentially counteract the medio-lateral parallel fiber dispersion of endorgan signals by preferentially making synapses on overlying Purkinje cells (31, 32). However, the density of synapses made by ascending granule cell axons on Purkinje cells is small (≤ 50) relative to the total number of synapses made by parallel fibers, ~200,000, as they pass through the Purkinje cell dendritic tree (27, 33). Furthermore, the amplitude of the EPSP evoked in Purkinje cells by selective activation of an ascending axon is no greater than that of the EPSP evoked by activation of a single conventional parallel fiber (30).

### Vestibular Secondary Mossy Fiber Afferents Terminate Bilaterally in the Cerebellum

In contrast to the ipsilateral projections of vestibular primary afferents, vestibular secondary afferents have bilateral projections from the DVN, MVN and SVN to vermal lobules IX-X and hemispheric lobule X (34–38). Vestibular secondary afferent mossy fibers terminate in more than one lobule and spread medially and laterally as they enter the granule cell layers of lobules IX-X in a pattern that mimics MFTs from the lateral reticular nucleus (Figure 1E) (9). The widespread distribution of MFTs onto granule cells contrasts with the narrow sagittal array of climbing fiber projections to Purkinje cell dendrites in lobules IX-X (Figure 1F).

The projection pattern of secondary vestibular afferent mossy fibers is bilateral and not restricted to lobules IX-X. Secondary vestibular afferents from the DVN and MVN also project to lobule VIII, the anterior vermis and paraflocculus (38, 39). Most of these ascending projections are cholinergic (40–43).

Purkinje cells in left vermal lobule X receive vestibular primary afferent mossy fiber projections that originate from the left vestibular endorgans. These same Purkinje cells receive vestibular climbing fiber projections that convey vestibular signals from the right inferior olive and right vestibular endorgans. The separate peripheral origins of vestibular climbing and mossy fibers influences how their signals modulate the activity of Purkinje cells.

### Vestibular Primary Afferents Project to GABAergic Neurons in the Parasolitary Nucleus

**Figure 2:**
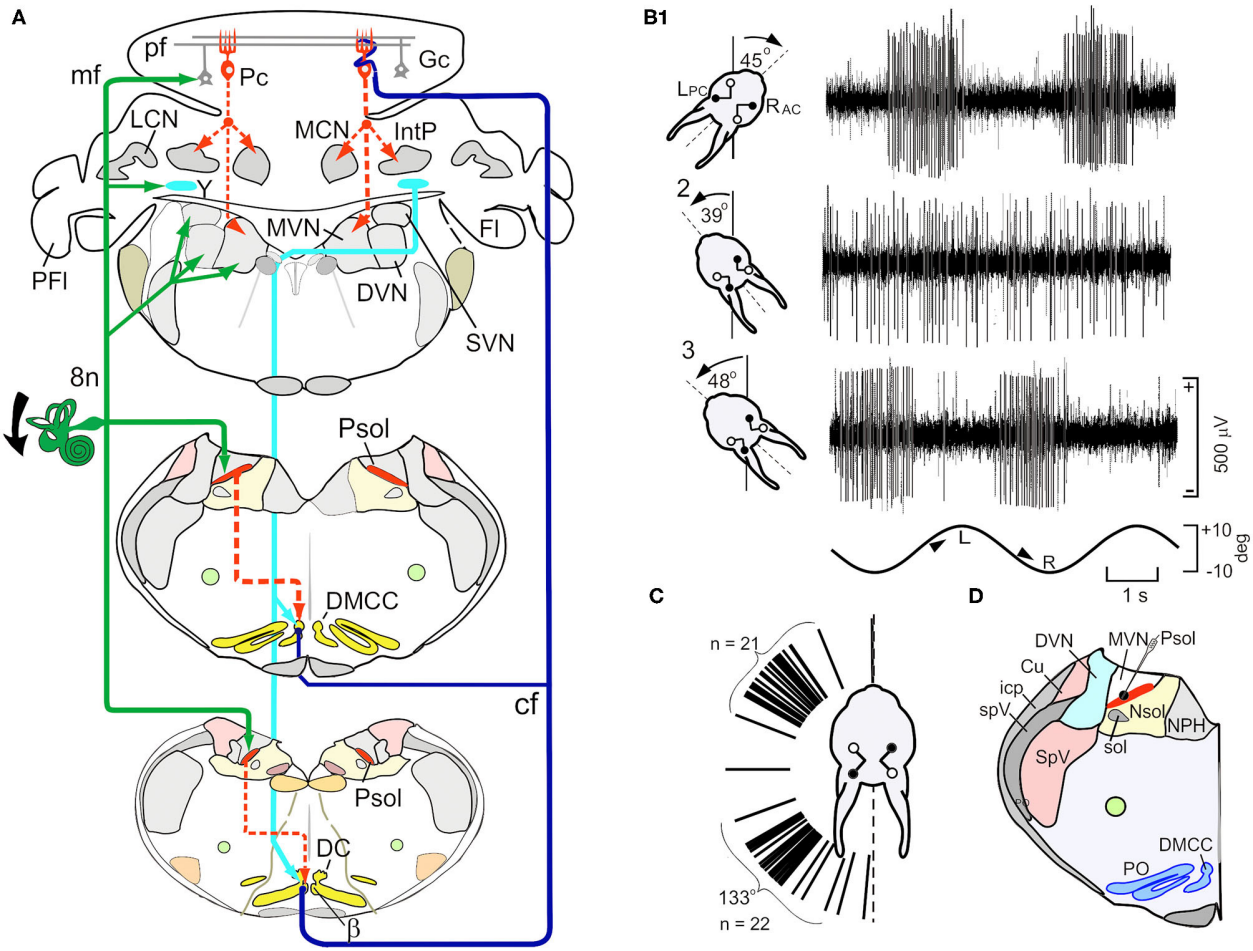
Schematic paths of vestibular mossy and climbing fiber and activity of neurons in the parasolitary nucleus (Psol). **(A)** Schematic illustrates the vestibular mossy and climbing fiber projections to the brainstem and posterior cerebellar cortex. Vestibular primary afferent mossy fibers (mf) (green lines) project to the ipsilateral Psol, medial, descending, superior vestibular nuclei (MVN, DVN, and SVN), Y-group (Y) and cerebellar granule cells (Gc). GABAergic Psol neurons project to the ipsilateral β-nucleus (β) and dorsomedial cell column (DMCC) (dashed red lines). Neurons in the β and DMCC project as climbing fibers (cf) to contralateral lobules VIII-X (dark blue lines). Y-group neurons project to contralateral DC, β and DMCC (light blue lines). **(B1)** Vestibular stimulation about the longitudinal axis modulates the activity of a neuron in the left Psol. During leftward roll-tilt, discharge is modulated best when the left posterior semicircular canal is oriented 45 deg clockwise (CW). The sinusoidal rotation is indicated at the bottom of the panel. **(B2)** When the head of the rabbit is oriented 39 deg counter clockwise (CCW), neuronal discharge is not modulated, defining a null plane in which the axis of rotation of the LPC is orthogonal to the longitudinal axis. (A3) When the head of the rabbit is oriented 48 deg CCW with respect to the longitudinal axis (~9 deg past the null plane), modulation of neuronal activity again appears and is phase shifted by 180 deg with respect to the sinusoidal stimulus. **(C)** Optimal response planes for 47 Psol neurons cluster about the anatomical orientation of either the ipsilateral anterior or posterior semicircular canals. **(D)** The neuron illustrated in **(A)** is localized to Psol. Bg, Bergman astrocyte; Cu, cuneate nucleus; Fl, flocculus; Gc, granule cell; Go, Golgi cell; icp, inferior cerebellar peduncle; IntP, interpositus nucleus; LCN and MCN, lateral and medial cerebellar nucleus; Lu, Lugaro cell; NG2^+^, glial cell; pf, parallel fiber; Pc, Purkinje cell; PFl, paraflocculus; Psol, parasolitary nucleus; 8n, vestibular nerve; MVN and DVN, medial and descending vestibular nuclei; NPH, nucleus prepositus hypoglossi; Nsol, nucleus solitarius; PO, principle olive; Psol, parasolitary nucleus; spV, spinal trigeminal tract; SpV, spinal trigeminal nucleus. Modified from Barmack and Yakhnitsa (14).

Psol is a cluster of small, cells compacted between the caudal MVN, DVN and solitary nucleus. They are uniformly GABAergic and project to the β-nucleus and dorsomedial cell column (DMCC) of the ipsilateral inferior olive (Figures 2A,D) (44, 45). The activity of single Psol neurons is modulated by vestibular stimulation during rotation in a three-axis rate table. The modulated activity increases during ipsilateral roll-tilt and decreases during contralateral roll-tilt. By altering the angle of the head with respect to the axis of rotation it is possible to find the plane of rotation at which the modulated activity of a recorded neuron is optimal (Figures 2B,C). Psol neurons are unresponsive to rotation about the vertical axis (13). Optimal response planes of Psol neurons are distributed throughout the hemifield, but align primarily with the anterior or posterior semicircular canals (Figure 2C).

### Vestibular Primary and Secondary Afferents Project to the Dorsal Y-Group

Psol is one of two pre-olivary nuclei that provides vestibular inputs to the β-nucleus and DMCC. The other originates from the Dorsal Y-group. The ventral Y-group projects to the flocculus (46). Primary vestibular afferents and projections from vestibular nuclei terminate on Dorsal Y-group neurons which, in turn, project to the ipsilateral flocculus and nodulus, contralateral oculomotor complex and inferior olive (17, 47–51). Neurons from the Dorsal Y-group descend and cross the midline to terminate in the contralateral β-nucleus, DMCC and rostral DC. Dorsal Y-group neurons are immunolabeled by an antibody to aspartate and are excitatory (52).

### Inferior Olivary Neurons Receive a GABAergic Projection From Psol Neurons

**Figure 3:**
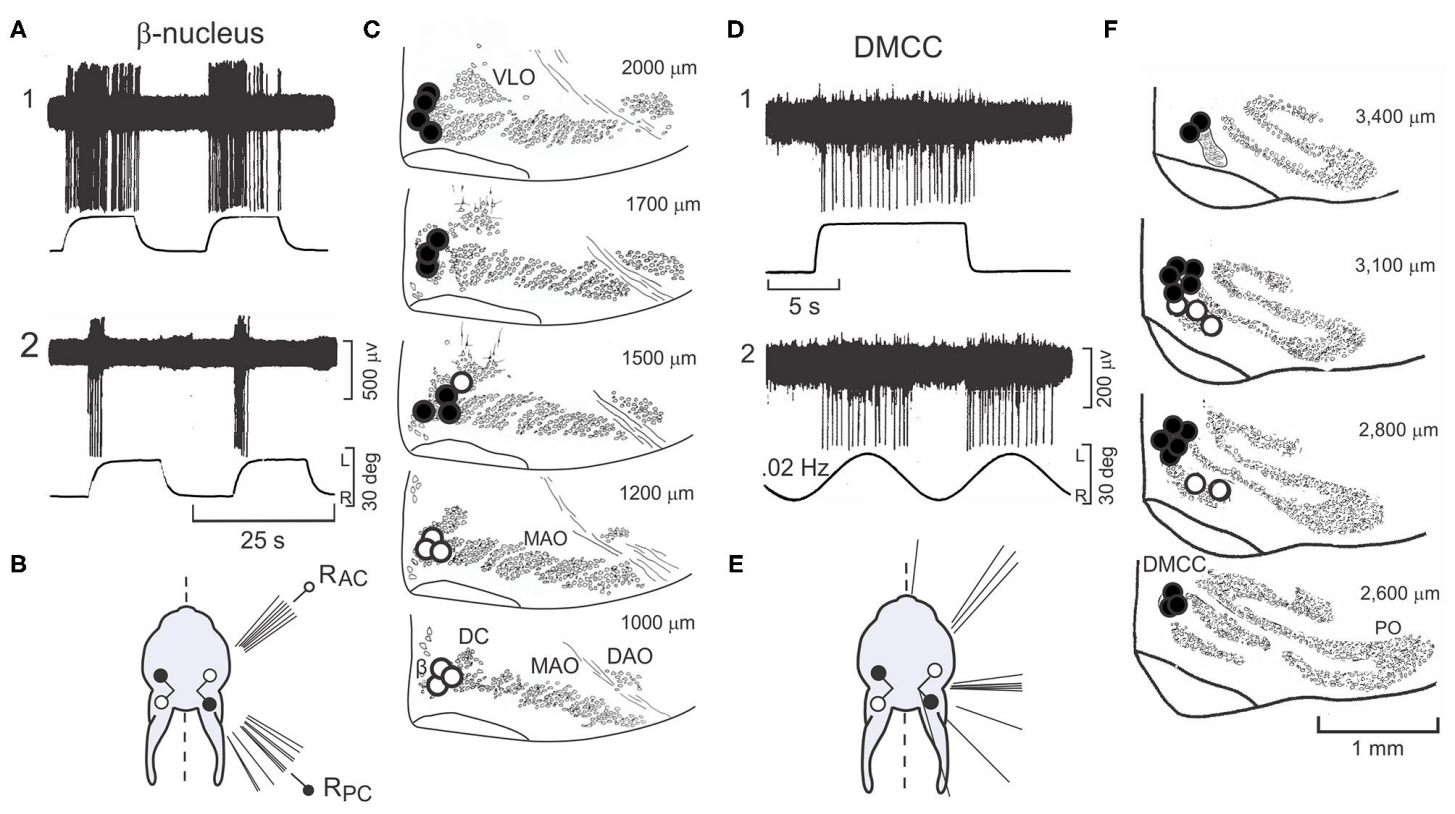
Neurons in β-nucleus and Dorsomedial Cell Column respond to step-roll about the longitudinal axis. Contralateral roll-tilt increases discharge in neurons in the β-nucleus and dorsomedial cell column (DMCC) respond to. **(A)** The responses of two neurons recorded from the right β-nucleus to step-roll stimulation differ. The first responds to velocity and position during step-roll onto the left side. The second, responds primarily to velocity. **(B)** The figurine illustrates the distribution of optimal response planes for 15 neurons recorded from β-nucleus. While some respond to static head position, the optimal response planes of all the neurons aligned with the anatomical planes of either the RAC or RPC. **(C)** Neurons in the β-nucleus are mapped onto transverse sections of the olive. Optimal response planes that align with the RAC are located in the caudal β-nucleus (open circles). Neurons with optimal response planes that align with the RPC are found more rostrally (filled circles). The numbers to the right of each panel indicate distance from the caudal pole of the inferior olive. **(D)** Two neurons recorded from the right DMCC respond primarily to head position. **(E)** The figurine illustrates the distribution of optimal response planes for 19 neurons recorded from DMCC. **(F)** Neurons with optimal response planes that align with the vertical canals are distributed throughout the rostro-caudal extent of the DMCC. Numbers in the upper right corner indicate the distance of each transverse section from the caudal pole of the inferior olive. Modified from Barmack et al. (45). DAO, dorsal accessory olive; DC, dorsal cap; DMCC, dorsomedial cell column; MAO, medial accessory olive; PO, principal olive; RPC, right posterior semicircular canal; VLO, ventrolateral outgrowth.

Two olivary nuclei, the β-nucleus and DMCC, receive descending inhibitory projections from the ipsilateral Psol (13, 45). Sinusoidal roll-tilt about the longitudinal axis modulates the discharge of neurons in the β-nucleus and DMCC. Roll-tilt onto the side contralateral to the recording site increases the discharge rate, and roll-tilt onto the ipsilateral side decreases it. This contralateral responsiveness of neurons in the β-nucleus and DMCC is consistent with the GABAergic projection from Psol. Like Psol neurons, β-nucleus and DMCC neurons have both static and dynamic sensitivity and are unresponsive to rotations about the vertical axis (Figures 3A1,2,B). The optimal response planes of neurons in the β-nucleus are topographically organized. β-nucleus neurons that align with the ipsilateral (right) anterior semicircular canal (RAC) are located caudally and neurons that align with the ipsilateral (right) posterior semicircular canal (RPC) are located rostrally (filled circles) (Figure 3C).

Like the β-nucleus, the DMCC receives descending GABAergic projections from Psol (Figure 2A). Neurons in the DMCC have optimal response planes distributed throughout the contralateral hemifield indicative of a more pervasive otolithic input (13, 53) (Figures 3D1,2E,F).

### Climbing Fibers Are Aligned in Sagittal Zones in Vermal Lobules IX-X

Neurons in the β-nucleus and DMCC project contralaterally to Purkinje cells in vermal lobules IX-X in narrow sagittal zones (9, 54–61). As they enter the cerebellum climbing fibers branch sagittally to synapse upon ~7 Purkinje cells (rat) within a multi-folial sagittal zone (62). A single climbing fiber makes ~500 synaptic contacts (rat) as it entwines Purkinje cell proximal dendrites (63).

**Figure 4:**
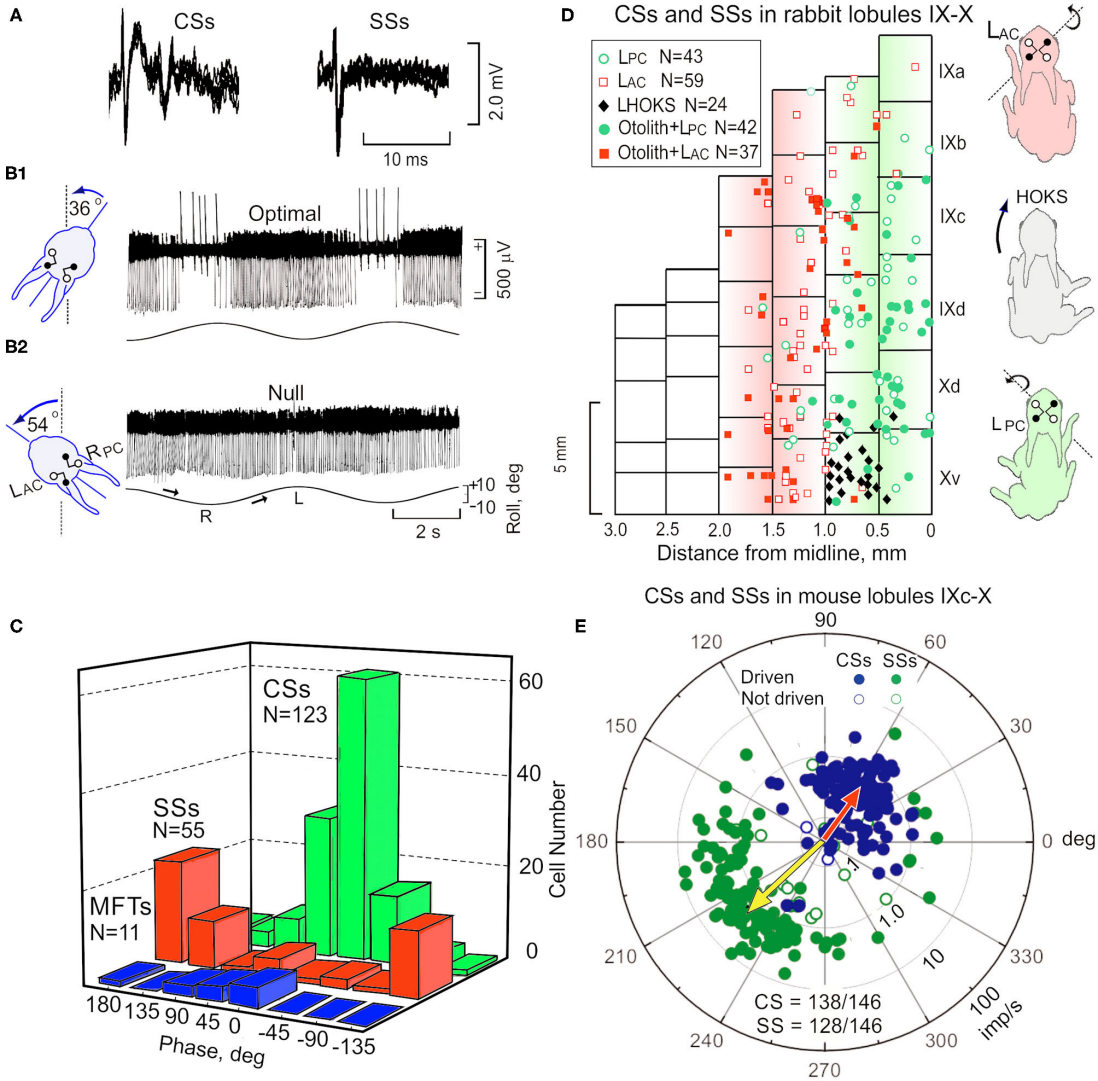
Sinusoidal roll-tilt modulates the discharge of CSs, SSs and MFTs in lobules IX-X rabbit and mouse. **(A)** CSs are discriminated from SSs on the basis of their multi-peaked action potentials of longer duration. Five superimposed traces for each waveform are shown. **(B1)** Sinusoidal roll-tilt modulates the discharge of CSs and SSs. CSs have are positive-going and SSs have negative-going action potentials. With the head maintained at a CW angle of 36 deg (see figurine), the axes of LAC and R_PC_ are aligned with the longitudinal axis of rotation and optimal antiphasic modulation of CSs and SSs is achieved. **(B2)** When the head angle is maintained at a CCW angle of 54 deg, the axes of the L_PC_ and R_AC_ a null plane is reached at which modulated of both CSs and SSs is minimal. **(C)** Histograms compare the phase and numbers of recorded CSs (green), SSs (red), and MFTs (blue) during sinusoidal roll-tilt. **(D)** The anatomical location of 205 Purkinje cells in rabbit cerebellum are plotted on a two-dimensional representation of lobules IX-X. Cells with optimal planes that are co-planar with the L_PC_ are green. Cells with optimal plane co-planar with the L_AC_ are illustrated as red squares. Open symbols indicate cells in which the optimal plane is not tested for otolithic responses. Filled symbols indicate cells tested for static sensitivity and are positive. Black diamonds indicate cells that are not responsive to vestibular stimulation, but are modulated by HOKS of the ipsilateral eye in the P →A direction. Figurines illustrate postural responses induced by vestibular and optokinetic stimulation in different planes. Vestibular stimulation of LAC evokes a lateral and forward extension of the ipsilateral fore- and hind-paws. P →A HOKS of the left eye evokes a lateral extension of the contralateral paws. Vestibular stimulation of the LPc evokes a backward extension of the ipsilateral paws. **(E)** Polar plot for 146 Purkinje cells in mouse cerebellum illustrates vectors and phases of CSs (blue) and SSs (green). Population vector for CSs is red and for SSs is yellow. gl, granule cell layer; ml, molecular layer; Pl, Purkinje cell layer. Modified from Barmack and Yakhnitsa (60), Barmack and Shojaku (64), Barmack and Yakhnitsa (65), and Barmack and Yakhnitsa (66).

Purkinje cells have two action potentials, termed Complex and Simple Spikes (CSs, SSs). CSs are multi-peaked action potentials and discharge at a rate of 0.1–5.0 imp/s. In contrast to the long duration and infrequent discharge of CSs, SSs are single peaked short duration action potentials (0.75-1.25 ms) that discharge at 20–60 imp/s (Figure 4A). Not surprisingly the activity of ~90% of the Purkinje cells recorded in vermal lobules IX-X is modulated by sinusoidal roll-tilt (59, 64, 67) (Figures 4A,B1,2). Consistent with the crossed projection of climbing fibers, the discharge of CSs increases during *ipsilateral* side-down rotation rather than *contralateral* side-down rotation characteristic of cells in the inferior olive. Null and optimal planes disclose the origin within both labyrinths of the modulated signal (Figures 4B1,2). The discharge for populations of CSs and MFTs with respect to the sinusoidal vestibular stimulation are similar. Both CSs and MFTs discharge maximally during ipsilateral side-down roll-tilt. By contrast SSs discharge maximally during roll-tilt onto the contralateral side, 180 deg out of phase with climbing and mossy fiber inputs (Figure 4C). These data make problematic the idea that mossy fibers convey the signal that modulates the discharge of SSs since the population mossy fiber signal leads the discharge of SSs by ~160 deg.

The response planes of vestibularly-driven CSs and SSs differ in Purkinje cells depending on the distance of Purkinje cells from the midline of vermal lobules IX-X. A sagittal strip of Purkinje cells, left of the midline, respond optimally during to ipsilateral roll-tilt when the head is maintained at an angle in which the axis of the L_PC_ and R_AC_ align with the longitudinal axis of rotation. In a second sagittal strip, lateral to the medial strip, Purkinje cells respond optimally during to ipsilateral roll-tilt in which the axis of the L_AC_ and R_PC_ align with the longitudinal axis of rotation. A two-dimensional representation of vermal lobules IX-X shows 146 Purkinje cells with optimal planes aligned with either the L_PC_ (green circles) or the L_AC_ (red squares) (Figure 4D). These two sagittal zones are ~800 μm wide in the rabbit. Within either the medial or lateral the amplitude of modulation for both CSs and SSs is maximal at the center of the zone and decreases in either the medial or lateral direction (59). The borders of the two zones overlap. Postural responses are influenced by vestibular stimulation about three different axes. Vestibular stimulation about the rotational axis of the L_AC_ evokes a forward and lateral extension of the ipsilateral fore- and hind-paws. Vestibular stimulation about the rotational axis of the L_PC_ evokes a backward extension of the left paws. These two dimensions are insufficient to maintain balance in a three-dimensional space. Rotation about a third, vertical axis is detected by a horizontal optokinetic signal that originates from the contralateral dorsal cap of Kooy (DC) (68). The climbing fibers that originate from the DC respond optimally to posterior→anterior (P→A) stimulation of the ipsilateral eye (54, 64, 69, 70). In rabbits, an optokinetic parasagittal climbing fiber zone of Purkinje cells is interposed between the L_PC_ and L_AC_ zones on the ventral surface of left vermal lobule X. This horizontal optokinetic zone completes the third spatial dimension needed for three-dimensional balance (1, 54, 64) (Figure 4D).

In the mouse cerebellum, sagittal zones for optimal planes of SSs and CSs are consistent with the zones found in rabbit. In the mouse, these physiologically defined zones are ~400 μm wide. The optimal planes of CSs and SSs can be quantified separately and displayed as a polar vector in which the amplitude of the vector corresponds to depth of modulation (**M**) and the phase angle (**ϕ**) corresponds to the phase of the response relative to the head position. For example, when ϕ = 0 deg, the peak discharge of a Purkinje cell in the left nodulus is in phase with maximal tilt of the head onto the left side (Figure 4E). When ϕ = 180 deg, the peak Purkinje cell discharge is in phase with peak rightward head tilt. Note that the population vector phase for CSs, ϕ = 56 deg, leads head position. The population vector for SSs, ϕ = 222 deg, lags CSs by 166 deg).

### A Three-Axis Floccular Optokinetic Coordinate System Is Composed With Climbing Fiber Signals

Vermal lobule X comprises a hybrid three axis coordinate system that uses signals from the utricular otolith, vertical semicircular canals and a horizontal optokinetic pathway. Three distinct parasagittal climbing fiber zones demarcate the separate termination of these inputs. By contrast the hemispheres of lobule X comprise a three-axis system that uses optokinetic information conveyed by climbing fibers to track head position in three-dimensional visual space. The coordinates of this three-dimensional system roughly align with the orientation of semicircular canals and utricular otoliths also used by lobules IX-X.

Hemispheric lobule X receives a visual projection from the DC. The DC receives an optokinetic signal from the ipsilateral nucleus of the optic tract (NOT) and projects to the contralateral hemispheric and vermal lobule X (54, 68, 71–73). While more than 90% of the mossy fiber projections to vermal lobules IX-X are vestibular primary afferent collaterals, the mossy fiber projections to hemispheric lobule X are heterogeneous. The largest mossy fiber projections to the flocculus originate from the dorsomedial medullary reticular formation, paramedian reticular nucleus and the nuclei of the paramedian tracts as well as the raphe nuclei (74).

**Figure 5:**
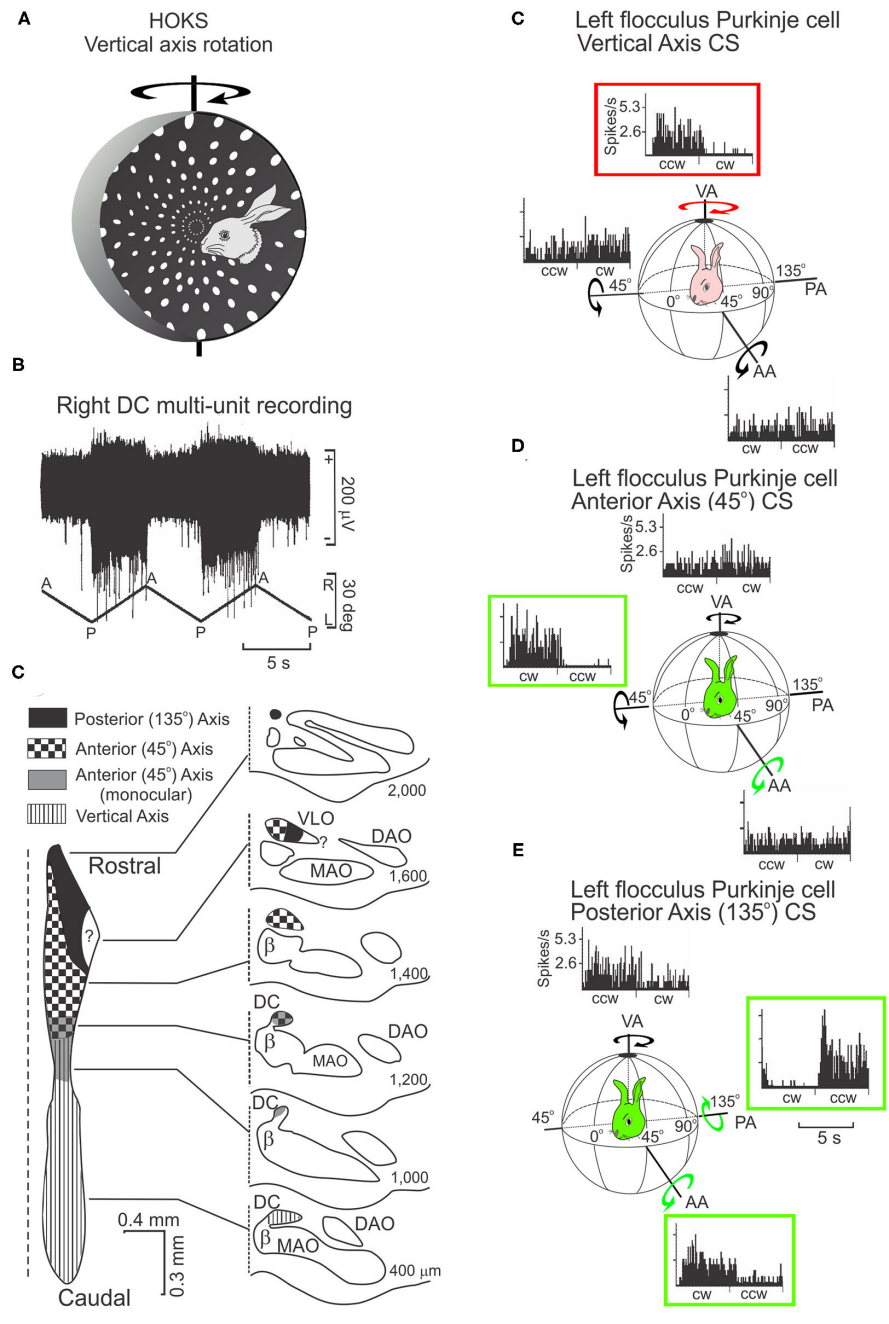
HOKS evokes discharge of neurons in the dorsal cap (DC) and discharge of Purkinje neurons in the flocculus. **(A)** HOKS about the vertical axis (VA) modulates the activity of caudal DC neurons. **(B)** Activity of multiple neurons in the caudal, left DC increases during low velocity (1 deg/s) HOKS in the posterior→anterior (P→A) direction of the right eye. The neuronal activity is disfacilitated during A→P stimulation. **(C)** Inferior olive is divided into regions corresponding to activity modulated by optokinetic stimulation about one of three axes (AA, PA, VA). An unfolded longitudinal strip of DC neurons is illustrated to the left, transverse sections to the right. The numbers to the right of each transverse illustration show the distance of the section from the caudal pole of the inferior olive. **(C)** P→A HOKS of the ipsilateral eye about the VA, increases the frequency of CSs. The discharge frequency of CSs is not modulated by OKS about the AA or PA. **(D)** The discharge of CSs in a second Purkinje cell is modulated by OKS of the contralateral eye in the CW direction about the AA. **(E)** The discharge of a CSs in a third Purkinje cell is modulated by OKS in the CCW direction about the PA. All recordings are obtained from the left flocculus. bp, brachium pontis; fpl, primary floccular fissure; Fl, flocculus. Modified from Barmack and Hess (75) and Leonard et al. (76).

Neurons in the caudal DC respond to P→A horizontal optokinetic stimulation (HOKS) of the contralateral eye (Figures 5A,B). The peak sensitivity to HOKS is 1 deg/s. The velocity sensitivity of the optokinetic response is reduced by 3 dB at 0.1 deg/s and 10.0 deg/s (75). Two other clusters of neurons in the DC convey information concerning OKS about the posterior axis (PA) and anterior axis (AA) (Figures 5C–E). These two axes lie along the azimuth and form angles of 135 deg (PA) and 45 deg (AA) with respect to the longitudinal axis (3, 69, 76). Each cluster in the DC projects to a sagittally arrayed zone in the contralateral flocculus (77, 78).

Vestibular and optokinetic climbing fiber systems in lobules IX-X have a similar organizational structure at the inferior olive and cerebellar cortex. Both systems are organized in three-dimensions. The vestibular system, anchored anatomically in the β-nucleus and DMCC, maintains a true gravitational vertical reference. However, it lacks input from the horizontal semicircular canals for detection of head rotation about the vertical axis. Instead, it relies on a small horizontal optokinetic input from the DC. The optokinetic system, anchored anatomically in the DC, detects self-motion about three axes, but lacks the non-visual dynamic and static sense of self-motion provided by the three semicircular canal ampullae and two otolith maculae.

The distribution of vestibular mossy fiber and vestibular climbing fiber signals assures that information from both labyrinths is represented bilaterally. Purkinje cells in left vermal lobule X (nodulus) receive vestibular primary afferent mossy fiber projections that arise from the left vestibular endorgans. These same Purkinje cells receive vestibular climbing fiber projections that convey vestibular signals from the contralateral inferior olive and right vestibular endorgans.

### Floccular Optokinetic Zones Are Demarcated Anatomically and Physiologically

The flocculus, like the uvula-nodulus, has a topographic spatial map represented in its climbing fiber projection. The rabbit flocculus has five transfolial zones (1, 2, 3, 4, and C2) whose borders can be defined both anatomically and physiologically. Anatomically the zone borders in the floccular white matter can be visualized by a histochemical stain for acetylcholinesterase (AChE) (2, 57, 79–81). Climbing fibers from the caudal DC encode HOKS about the vertical axis (VA) to zones 2 and 4. Cells in rostral DC and ventrolateral outgrowth (VLO) detect OKS about the posterior axis (PA) and anterior axis (AA) and project to zones 1 and 3. These two axes lie along the azimuth and form angles of 135 deg (PA) and 45 deg (AA) with respect to the longitudinal axis. Climbing fibers from the rostral medial accessory olive (MAO) project to zone C2, but convey no optokinetic information (57, 69, 78, 82). Climbing fibers from the rostral DC in the rabbit encode optokinetic stimulation about the PA and AA and project to zones 1 and 3. Tracer and micro-stimulation studies suggest that these floccular zones have different projection patterns particularly to the vestibular complex (57, 81). Microstimulation of the white matter in the rabbit flocculus evokes eye movements consistent with the zone that is stimulated. Microstimulation of zones 2 and 4 evoke horizontal eye movements. Microstimulation of zones 1 and 3 evoke movements about the AA and PA axes (2). The function of the C_2_ compartment may be linked to the control of head movement (83).

### Immunohistochemical Zebrin II Parasagittal Zones and Physiological Climbing Fiber Zones in Lobule X

Physiologically defined parasagittal climbing fiber zones can be compared to the parasagittal zones described immunohistochemically using antibodies to Zebrin I and Zebrin II (84, 85) and to histochemical stains for acetylcholinesterase (86) and 5′-nucleotidase (87, 88). The expression of Zebrin II in vermal lobule X intensely labels Purkinje cells with only weakly labeled interlaced Zebrin negative bands (85, 89, 90). The prominent interlaced immunohistochemical zonation is more apparent in vermal lobule IX and the hemisphere of lobule X (flocculus).

Purkinje cells in in lobule X of the pigeon respond to one of the three axes of optokinetic stimulation (91), similar to the physiological pattern of optokinetic responses observed in rabbit (69) and mouse (92). Within a physiological optokinetic zone both zebrin II positive and zebrin II negative Purkinje cells exist. The amplitude of modulated discharge for Purkinje cells located in a zebrin II-positive zone exceeds that of Purkinje cells located in a zebrin II-negative zone. The cause of this difference is unresolved. However, it seems that zebrin II zones are not congruent with the optokinetic and vestibular sagittal zones in lobules IX-X.

### The Antiphasic Discharge of CSs and SSs Depends Climbing Fibers

Presently, the origins of the synaptic signals that modulate the discharge of SSs is poorly understood. Often, it is argued that parallel fiber discharge modulates the activity SSs (93–102). If vestibular primary afferents→granule cells→parallel fibers→Purkinje cell were a straight-through pathway, then the discharge of vestibular primary afferents and the discharge of SSs ought to co-vary. However, during sinusoidal roll-tilt these two signals are antiphasic. SSs are also antiphasic with the discharge of CSs (Figures 4B,C,E) (64, 65). It is logically possible that vestibular nuclear neurons, a fraction of which project contralaterally, are the major synaptic driving force that modulates the activity of SSs, but at present there is no evidence that bears on this point. Perhaps the genesis of antiphasic discharge of CSs and SSs in lobules IX-X can be explained by known cerebellar circuitry.

An antiphasic interaction between CSs and SSs can be demonstrated directly by reversibly inactivating the inferior olive. Application of a cooling probe to the ventral brainstem of the rat reduces CS discharge and increases SS discharge (103, 104). The increased SS discharge reduces the spontaneous discharge rate of secondary vestibular neurons and cerebellar nuclear neurons. This cooling effect can be attributed to the decreased olivary activity. When the inferior olive is destroyed by a cocktail of 3-acetylpyridine and harmaline prior to cooling, the increased discharge of SSs evoked by cooling the inferior olive in intact rats does not occur (104–106).

The antiphasic discharge of CSs and SSs following activation of climbing fibers does not depend on the occurrence of a CS in the same Purkinje cell. When climbing fibers are electrically stimulated in the inferior olive, the intensity of the electrical stimulus can be adjusted to be above or below a level needed to evoke a CS in a recorded Purkinje cell. Suprathreshold electrical stimulation evokes a CS and also suppresses the spontaneous discharge of SSs. However, a stimulus that is subthreshold for activating a CS in a recorded Purkinje cell may still cause suppression of its SSs (107). This finding has been replicated, substituting optical stimulation of channel rhodopsin-expressing climbing fibers instead of electrical stimulation. In this instance the spontaneous activity of a Purkinje cell whose climbing fiber is not activated, nevertheless decreases (108). This supports the idea that the suppression is caused by climbing fiber-evoked activation of inhibitory interneurons.

The effects of CSs on the discharge of SSs can be examined by altering the wiring of climbing and mossy fiber projections to the cerebellum. In the native pathway climbing fibers decussate in the ventral brainstem and then project to the contralteral cerebellum. It is possible to mutate this climbing fiber pathway so that the normal crossed contralateral projection is converted to an uncrossed ipsilateral projection. This is accomplished by insertion of the mutation (Ptf1a::cre;Robo3(lox/lox) in mice (109). The normally uncrossed mossy fiber projections remain undisturbed. Purkinje cell recordings from the flocculus of these mutant mice during optokinetic stimulation indicate that the favored P→A directional preference of normal mice is reversed in mutant mice. The CS discharge is now optimal for stimulation in the A→P direction because the uncrossed olivo-cerebellar projection in the mutant. More interesting is that the directional preference of optokinetically-evoked SSs is also reversed in the mutant. If the modulated discharge of SSs was caused by an intact mossy fiber→granule cell→parallel fiber projection, its polarity would not reverse.

**Figure 6:**
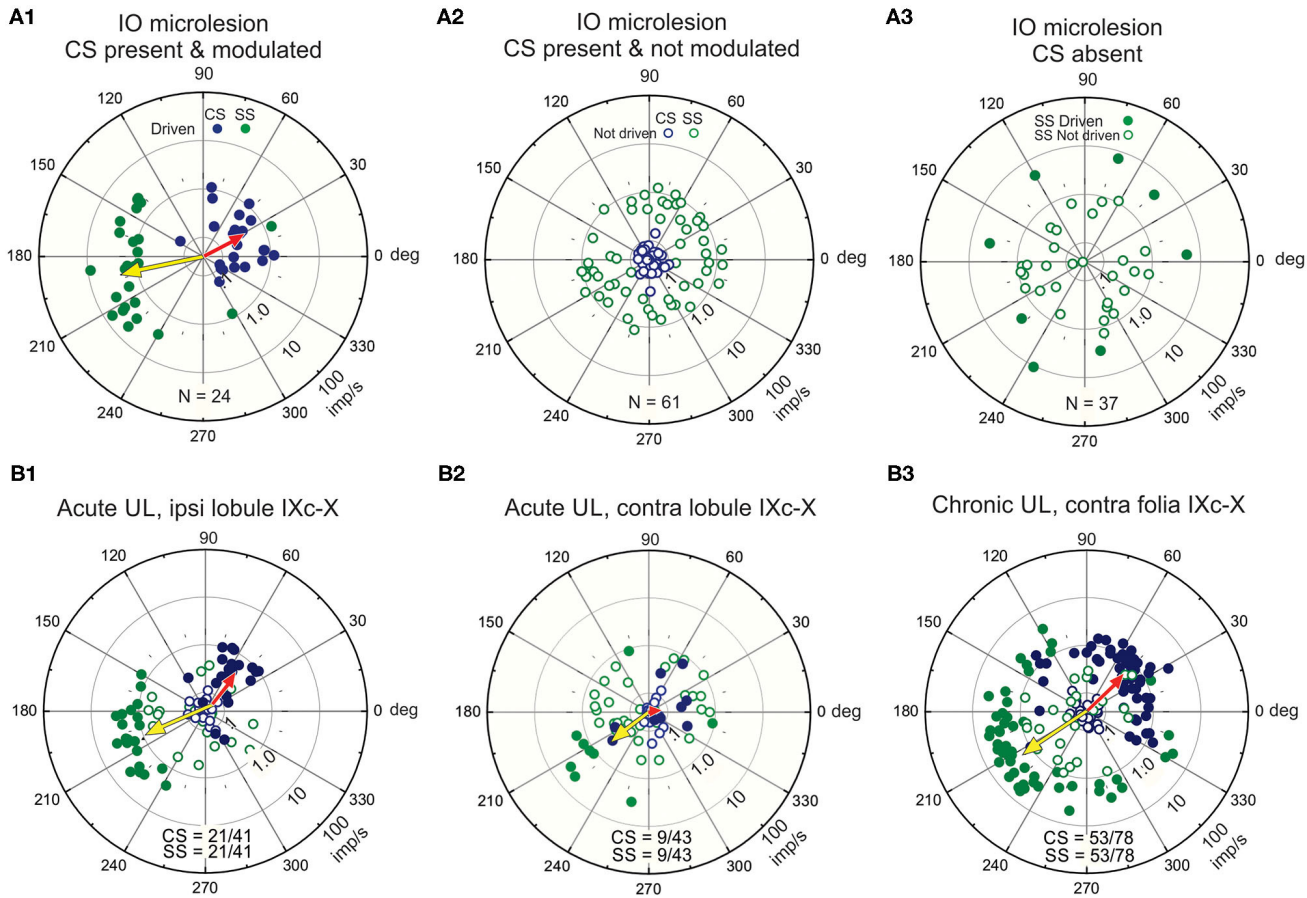
Unilateral microlesions of the β-nucleus and unilateral labyrinthectomy (UL) alter the antiphasic discharge of CSs and SSs in vermal lobule IXc-X Purkinje neurons. **(A1–3)** Incomplete, unilateral microlesions are made in the right β-nucleus to examine how the discharge of SSs in lobules IX-X Purkinje cells are modulated by sinusoidal vestibular stimulation about the longitudinal axis with and without climbing fiber signals. **(A1)** Microlesions are incomplete. Some Purkinje cells with intact climbing fibers evince an antiphasic discharge of CSs and SSs. A polar plot of these responses and their population vectors is similar to that characterizes Purkinje cell discharge in mice with totally intact climbing fiber projections (see Figure 4E). **(A2)** Some Purkinje cells retain spontaneous CSs, but the discharge of these CSs is no longer modulated by vestibular stimulation. In these instances, SSs are also spontaneously active, but no longer driven by vestibular stimulation. **(A3)** In a third group of Purkinje cells, the microlesion destroys the climbing fiber projection. In this group, the discharge of 30/37 SSs is not modulated by vestibular stimulation. **(B1–3)** A UL deprives the ipsilateral vermal lobules IX-X of a vestibularly modulated primary afferent mossy fiber signal while leaving the contralateral primary afferent mossy fiber signal to the contralateral vermal lobules IX-X intact. **(B1)** The proportion of Purkinje cells ipsilateral to the UL in which CSs and SSs are antiphasically modulated vestibular stimulation is reduced by (21/41), but population vectors are comparable to those in normal mice. (B2) The proportion of Purkinje cells contralateral to the UL in which CSs and SSs are antiphasically modulated is reduced (9/43) even though their primary afferent mossy fiber input remains intact. The population vector for SSs is similar to that of normal mice, but the population vector for CSs is reduced to 0. **(B3)** Recordings, made at least 48 h post-UL show recovery of CS-induced SS pauses in contralateral Purkinje cells. Modified from Barmack and Yakhnitsa (66, 110).

Purkinje cells in lobules IX-X receive an excitatory climbing fiber signal from the contralateral β-nucleus and DMCC (Figure 2B). If the climbing fiber input were blocked by making a microlesion in the right β-nucleus, then Purkinje and stellate cells in the left lobules IX-X would retain only a vestibular primary afferent signal while right vermal lobules IX-X would retain both a climbing fiber signal and a vestibular primary signal. The antiphasic discharge of CSs and SSs is disrupted following a microlesion of the right β-nucleus. Such a microlesion leaves some cells in the β-nucleus and DMCC and their climbing fiber projections to Purkinje cells in the contralateral vermal lobules IX-X intact. The CSs and SSs in these Purkinje cells respond to sinusoidal roll-tilt with a normal antiphasic discharge (Figure 6A1). A second group of Purkinje cells retain spontaneously discharging CSs, but they are not driven by vestibular stimulation. The SSs in these Purkinje cells are unresponsive in spite of the fact that their vestibular primary afferent mossy fiber input is not compromised (Figure 6A2). A third group of Purkinje cells lack spontaneous and vestibularly-modulated CSs. In these Purkinje cells, 30/37 SSs cannot be modulated by vestibular roll-tilt (Figure 6A3) (110).

Vestibular primary afferent mossy fibers project exclusively to ipsilateral vermal lobules IX-X. Following a left unilateral labyrinthectomy (UL) Purkinje cells recorded from ipsilateral (left) vermal lobules IX-X retain a normal climbing fiber-evoked antiphasic response of CSs and SSs even though the left vestibular primary afferent mossy fiber signal is effectively removed (Figure 6B1). The climbing fiber pathway to the left vermal lobules IX-X remains intact since it originates from the contralateral (right) labyrinth. Conversely, the antiphasic responses of CSs and SSs in the right vermal lobule IX-X Purkinje cells ipsilateral to the *intact* labyrinth, are severely impaired (66) because the climbing fiber input to the left vermal lobules IX-X originates from the surgically destroyed (right) labyrinth (Figure 6B2). These recordings are made acutely within 3 h post-UL. Chronic recordings, are made at least 48 h post-UL reveal a strong recovery of CS-induced SS pauses in contralateral Purkinje cells (Figure 6B3). This can be attributed, in part, to the recovery in the activity of secondary neurons that project bilaterally to vermal lobule X (38, 111).

### Cerebellar Interneurons and the Genesis of Climbing Fiber-Evoked Pauses

Climbing fiber evoked pauses in SS discharge can be viewed at three levels: [1] Spontaneous SS pauses are triggered for 5–10 ms due to the large climbing fiber-induced inactivation by a Ca2+ activated K+ conductance (112). A short-term SS inactivation may also be attributed, in part, to ephaptic coupling of Purkinje cells (113). This climbing fiber-evoked pause of SSs occurs even when GABAergic transmission by interneurons is blocked (114, 115). [2] Long-term depression (LTD), evoked by conjunctive activation of climbing fiber and parallel fiber synapse on a Purkinje cell dendrite has a duration of at least minutes (116–122). [3] A longer pause in SS discharge is induced by climbing fiber-evoked interneuronal inhibition. Three inhibitory interneurons are likely candidates to play this role; Golgi cells, stellate cells and basket cells. This pause is independent of the fast changes in Purkinje cell conductance and lasts 5-100 ms (123–126).

**Figure 7:**
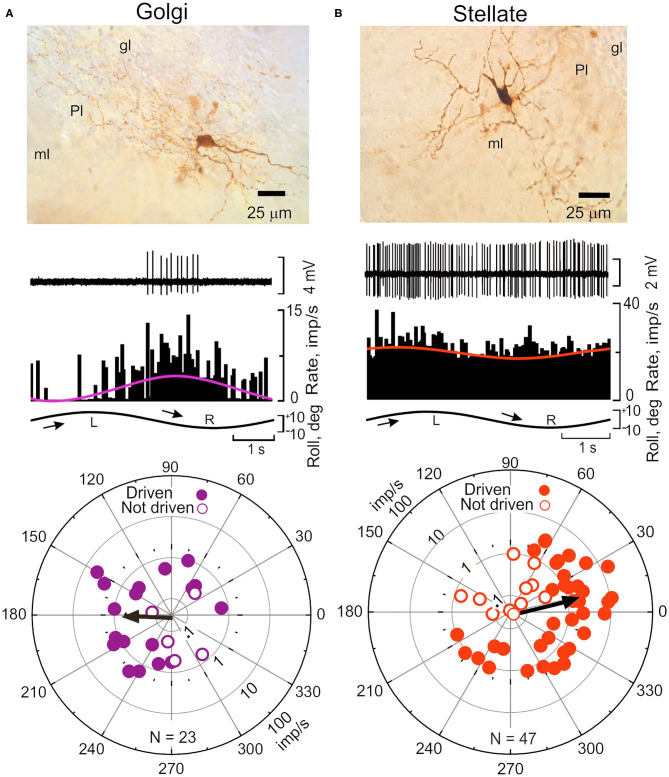
Sinusoidal roll-tilt modulates the discharge of Golgi and Stellate interneurons in lobules IX-X. **(A)** Interneurons are identified with juxtacellular labeling neurobiotin (138). **(A)** Golgi cell in the granule cell layer (gl) of left vermal lobule IX responds to *contralateral* roll-tilt. The peristimulus histogram is fitted with a cosine function (purple). The population vector for 23 Golgi cells, ϕ = 184 deg. **(B)** Stellate cell in left lobule X responds to *ipsilateral* roll-tilt. Peristimulus histogram is fitted with a cosine function (red). The population vector for 47 stellate cells, ϕ = 13 deg. gl, granule cell layer; ml, molecular layer; Pl, Purkinje cell layer. Modified from Barmack and Yakhnitsa (65).

Golgi cells have large somata (10–20 μm) found at the base of the Purkinje cell layer. Their dendrites are oriented sagittally in the molecular layer and have a planar width of 180 μm, comparable to the planar width of Purkinje cells, 120 μm (65). Golgi cell axons branch extensively in the granule cell layer where they co-terminate with mossy fiber terminals on granule cell dendrites in a glomerular plexus (6, 24, 127, 128). Golgi cell axon terminals also contact unipolar brush cells (129). Climbing fibers make synaptic contact with Golgi cell dendrites in the molecular layer (130, 131). Consequently, Golgi cells are in a unique position to regulate the discharge of granule cells and thereby influence parallel fiber input to Purkinje cells and possibly account for the modulation of SSs. The activity of Golgi cells can be recorded *in vivo* and juxtacellularly labeled to confirm subsequently their identity. The activity Golgi cells in lobules IX-X is modulated sinusoidal vestibular stimulation. Interestingly, Golgi cells are not driven in phase with either CSs or parallel fibers. Rather during sinusoidal roll-tilt they respond during contralateral side-down rotation, in phase with the discharge of SSs and out of phase with the discharge of CSs (ϕ = 180 deg), making it implausible that Golgi cells are responsible for the modulation of SSs during vestibular stimulation (65) (Figure 7A).

Stellate cell axons have lengths of ~250 μm and since they are arrayed throughout the entire molecular layer, make multiple and repeated contact with the dendrites of Purkinje cells (132, 133). The mouse molecular layer contains at least 15 stellate cells/Purkinje cell. Climbing fibers make no direct synaptic contacts onto either basket or stellate cells (128, 134). Rather, the discharge of climbing fibers releases glutamate (“spillover”) in sufficient concentration to excite stellate cell discharge (125, 126, 135–137). Stellate cell axon terminals release GABA onto GABA_A_α_1_ receptors on Purkinje cell dendrites.

The discharge of stellate cells is well-modulated during sinusoidal roll-tilt with peak discharge frequency during ipsilateral side-down. The polarization vector for a population of 47 stellate cells (ϕ = 13 deg) suggests that the antiphasic responses of CSs and SSs could be attribute to climbing fiber-evoked stellate cell inhibition of Purkinje neurons (Figure 7B) (65).

Stellate cell inhibition of Purkinje cells initiated by climbing fiber release of glutamate is not the only unconventional aspect of stellate cell synaptic influence on Purkinje cell excitability. If climbing fibers and parallel fibers are stimulated conjunctively in the cerebellar C3 zone of the cat, the stellate cell response to parallel fiber stimulation alone increases (139). Stellate cells express NMDA receptors. When stellate cells are stimulated in tissue slice preparations the stellate cells increase their discharge through a Ca2+ and CaMKII-dependent activation of voltage-gated Na+ channels (140). This signaling pathway lowers the gated action potential threshold by causing a hyperpolarizing shift in voltage gated Na+ channels. The increased discharge lasts for minutes.

### Oscillatory Adaptation of Vestibular Circuitry Occurs During Prolonged Roll-Tilt Stimulation

**Figure 8:**
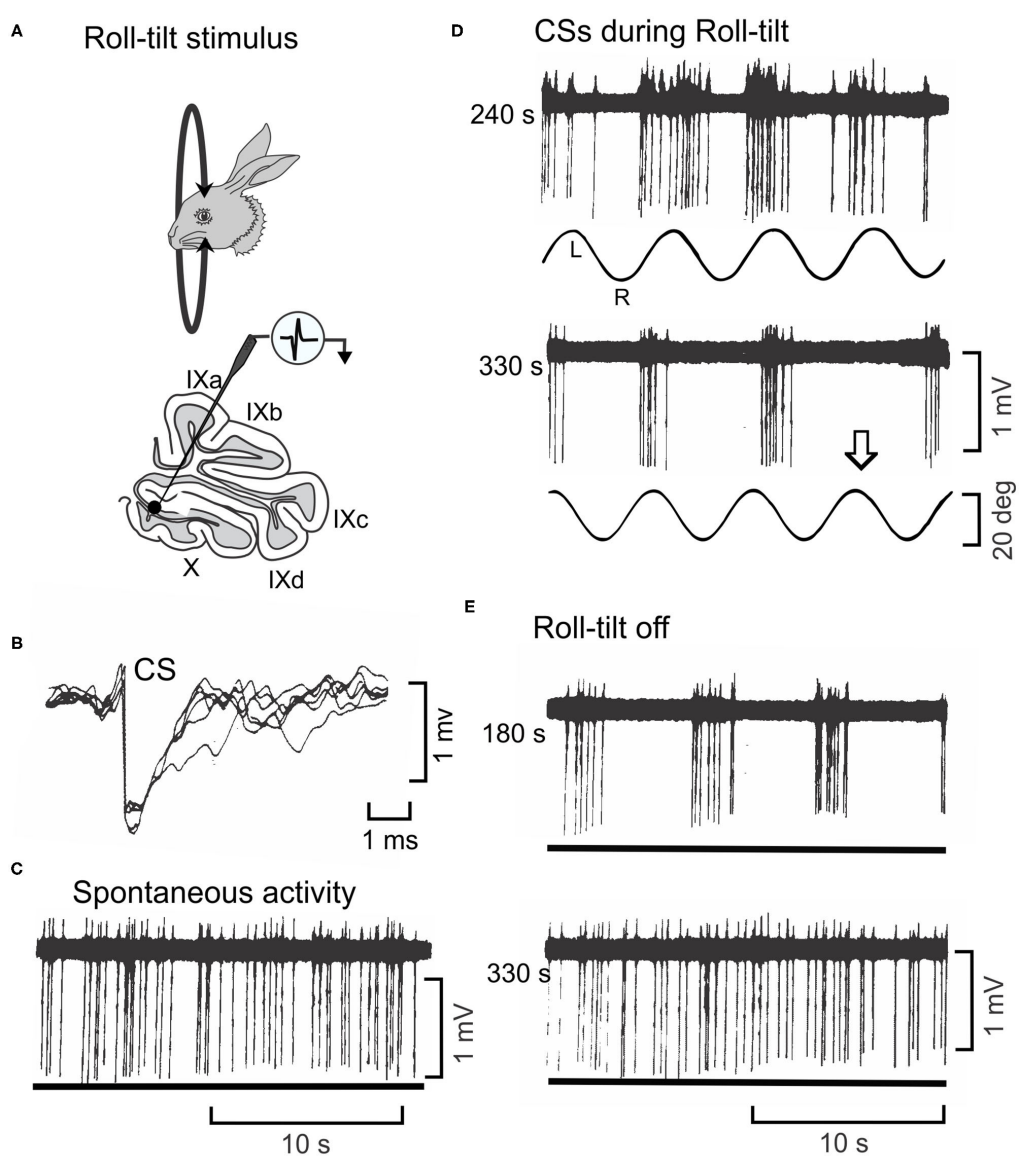
Oscillatory discharge of a CS cell in lobule X of rabbit is entrained by sinusoidal roll-tilt. **(A)** Figurines depict a rabbit during roll-tilt and a microelectrode recording from a Purkinje cell in the left lobule X. **(B)** Five superimposed waveforms identify a CS in recorded Purkinje cell. **(C)** Spontaneous discharge of CSs occurs in absence of roll-tilt stimulation (lower trace). **(D)** Sinusoidal roll-tilt continues to modulate discharge of CSs 240 s after the stimulation begins. CS discharge increases during roll-tilt onto the left side. After 330 s of continuous roll-tilt, the modulation CS discharge decreases. Arrow indicates one cycle during which no CSs occur. **(E)** When the vestibular stimulus stops, the discharge of CSs oscillates at the previous stimulus frequency. This oscillatory discharge still occurs 180 s after the vestibular stimulus stops. It abates after 330 s. Modified from Barmack and Shojaku (141).

The modulated pattern of discharge CSs in Purkinje cells during sinusoidal rotation about the longitudinal axis is usually invariant for tens of minutes (Figure 4B1). When vestibular stimulation stops, the pattern of CS discharge returns to a spontaneous level. However, the discharge of 5% CSs is not invariant (Figures 8A–E). In these Purkinje cells, the modulated discharge frequency of CSs decreases during repeated sinusoidal roll-tilt at 0.20 Hz (Figure 8D). After ~345 s the vestibular stimulus fails to evoke a CS discharge (141). When sinusoidal stimulation is discontinued, the oscillatory pattern of CSs at 0.20 Hz re-appears (Figure 8E). Approximately 200–300 s after vestibular stimulation stops, this pattern disappears and is replaced with aperiodic spontaneous discharge. This pattern of oscillations, can be temporarily entrained to a different frequency of roll-tilt. An oscillation at 0.20 Hz can be entrained to 0.30 Hz. When the stimulation stops, the entrained oscillation at 0.30 Hz rapidly fades into 0.20 Hz and then back to aperiodic spontaneous activity. While it is tempting to speculate that this adaptive pattern originates in the inferior olive, the same adaptive pattern can be observed in the GABAergic neurons in the Psol (14). Possibly the oscillations originate at the Psol or at a more peripheral level. The medial vestibular nucleus has class of secondary neurons that express N-Methyl-D-Aspartate receptors (NMDA) (142). In tissue slice experiments the discharge of these neurons oscillates at frequencies of 0.1–0.3 hz with bath application of NMDA (143). The possibility of NMDA receptors in neurons in the MVN and Psol acting similarly to the NMDA receptors in stellate cells has not been explored. Possibly NMDA receptors provide a mechanism by which membrane oscillations induced by vestibular stimulation are controlled by neuromodulators.

The oscillatory discharge of Psol neurons and climbing fibers, while present in anesthetized preparations, have a behavioral analog in unanesthetized rabbits. Sinusoidal linear acceleration of a rabbit along the inter-aural axis for several hours evokes oscillatory vertical eye movements. These eye movements persist for 1–2 min when vestibular stimulation is stopped (144).

### Activity of Floccular CSs Influences the Discharge of MVN Neurons

**Figure 9:**
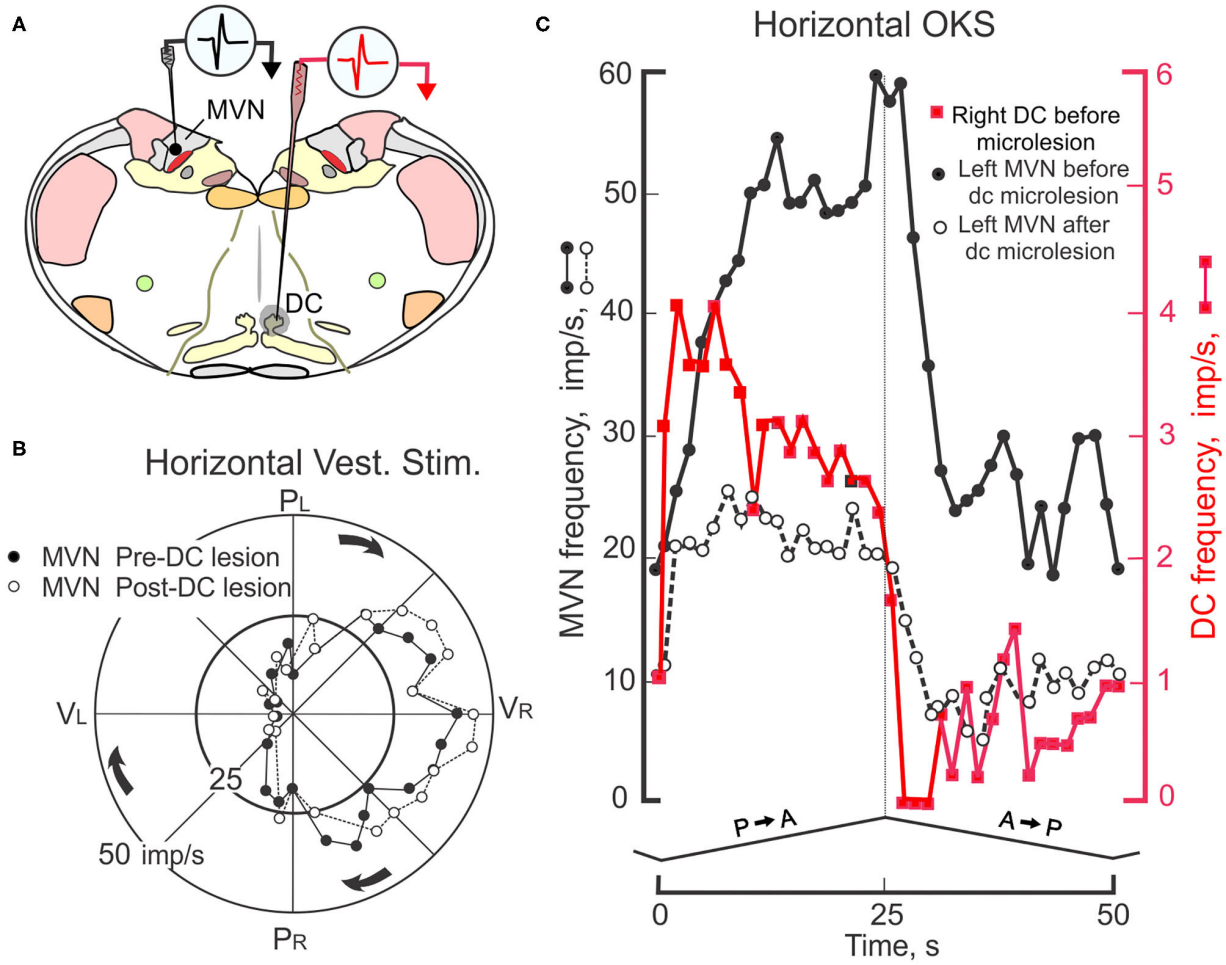
Interaction of floccular and non-floccular optokinetic signals at the level of the medial vestibular nucleus (MVN). **(A)** Transverse brainstem section shows locations for two electrodes used to record simultaneously from two neurons; one he left MVN and the other in the right caudal DC. The activity of this pair of neurons is recorded during four cycles of horizontal sinusoidal vestibular stimulation and four cycles of monocular, triangular waveform, constant velocity HOKS of 1 deg/s delivered to the left eye. A microlesion (-50 μa, 30 s) is then made at the recording site in the caudal DC. The destroyed area is indicated by shading. Subsequently, the responses of the MVN neuron during horizontal vestibular and HOKS are recorded again. **(B)** Polar plot shows MVN responses evoked during sinusoidal horizontal vestibular stimulation (± 10 deg, 0.4 Hz) in the dark before (filled circles) and after (open circles) the electrolytic microlesion is placed in the right caudal DC. The gain and phase of the discharge of the MVN neuron are unmodified by the DC microlesion. **(C)** HOKS modulates the activity of the left MVN neuron (black filled circles) and the right DC neuron (red filled squares). After the microlesion of the right DC, the optokinetically-evoked discharge of the left MVN neuron is reduced (open circles). Modified from Barmack (146).

MVN neurons receive optokinetic as well as vestibular signals (145). By recording from MVN neurons before and after the dorsal cap is removed from the optokinetic circuitry the contribution of the DC through the flocculus to visually-modulated MVN activity can be determined (Figure 9A). In paired recordings, both DC and MVN neurons increase their rate of discharge during constant velocity (0.8 deg/sec) of the eye ipsilateral to the MVN and contralateral to the DC (146). At the onset of P→A stimulation the MVN neuron slowly increases its rate of discharge from ~10 imp/s to a maximum of ~60 imp/s over the 25 s interval (Figure 9C). When the optokinetic stimulus reverses direction, the activity of the MVN neuron slowly returns to a steady-state with a 10–15 s decay. When the site of the olivary recording is inactivated by an electrolytic microlesion made through the olivary recording electrode, the response profile of the MVN neuron is altered. The peak modulation is decreases and the slow build up and decay attenuates. The DC microlesion has no effect on MVN activity induced by rotation of the rabbit about the vertical axis (Figure 9B). Consistent with the known circuitry, the optokinetically evoked activity of neurons in the right DC project as climbing fibers to Purkinje cells in the left (contralateral) hemispheric lobule X. The increase in climbing fiber-evoked CSs reduces the discharge of SSs in Purkinje cells, thereby withdrawing Purkinje cell inhibition of the subjacent MVN neurons.

### Hemispheric Lobule X Contributes Optokinetic Stabilization to Postural Control

In monkeys, post-rotatory vestibular nystagmus (PRN) is provoked by deceleration following constant velocity horizontal vestibular stimulation. PRN lasts for only a few seconds, but long enough to measure how the plane of PRN changes during pitch. Since monkeys have frontally placed eyes changes in pitch would be expected to alter the plane of PRN within the orbit to keep the plane in space constant. In normal monkeys, this is exactly what happens. In monkeys with bilateral lesions of the lobule X, PRN no longer is executed horizontally in space, but rather is executed horizontally within the orbit (147, 148).

**Figure 10:**
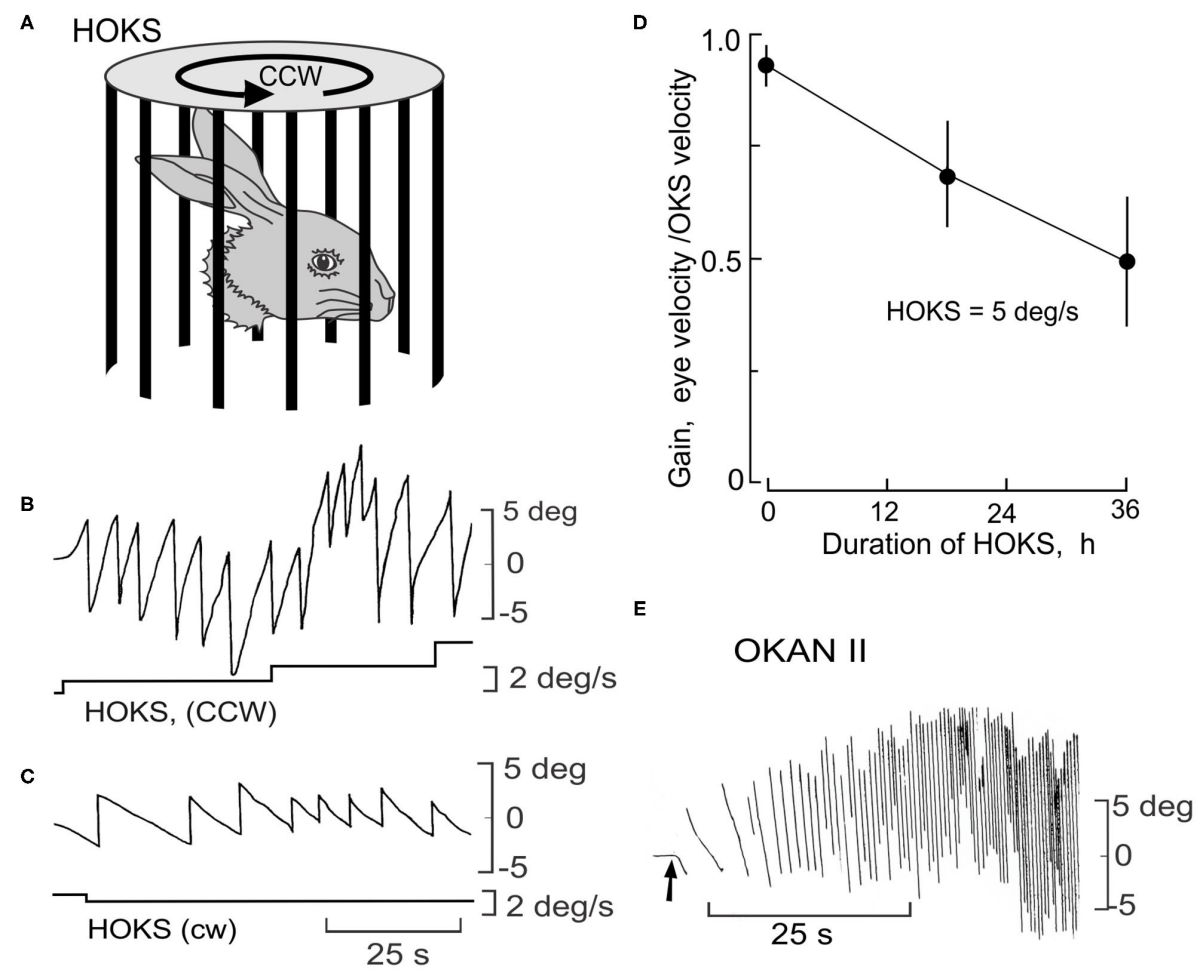
Long-term HOKS induces a prolonged negative optokinetic after-nystagmus (OKAN II). **(A)** Rabbits are exposed to monocular HOKS by placing them in the center of a cylinder that rotates at an angular velocity that changes stepwise from 1 to 5 deg/s and stimulates the right eye in the P→A direction. Eye position is measured with a photoelectric technique (149). Patterned vision of the left eye is occluded. **(B)** P→A HOKS of the right eye at 5 deg/s evokes horizontal eye movements with a slow phase velocity of ~4.5 deg/s. The bottom trace indicates HOKS velocity. **(C)** A→P HOKS of the right eye evokes an eye movement in the same direction, but with a lower velocity. **(D)** The gain of the eye movements evoked by binocular HOKS is measured in three rabbits at 0, 18, and 24 h after the onset of HOKS. The gain decreases with increasing duration of HOKS. **(E)** HOKS is maintained for 24 h. When it stops (arrow) and the rabbit is placed in the dark, the slow phase of the nystagmus reverses direction with respect to the now absent HOKS. OKAN II accelerates to a velocity of 30–50 deg/s and lasts for 12-24 h. Modified from Barmack and Nelson (150).

In rabbits the same influence of the gravitational vector and eye movements can be examined by inducing a long-lasting optokinetic after-nystagmus by providing sustained exposure to HOKS. In rabbits, sustained HOKS (1–48 h) evokes an optokinetic after-nystagmus (OKAN II) that lasts for several hours (Figures 10A–E). The velocities of the slow phase of OKAN II vary between 30 and 50 deg/s and exceed the normal range of rabbit slow phase velocities, even exceeding the slow phase velocities induced by a unilateral labyrinthectomy (Figure 10E). During sustained binocular HOKS the gain of the horizontal optokinetic reflex (eye velocity/drum velocity) decreases gradually from ~0.95 at the onset of stimulation to ~0.50 after 36 h of HOKS (Figure 10D). One way of thinking about the genesis of OKAN II is that the animal adapts to a visual environment that moves slowly about the vertical axis. Retinal slip increases as the gain of the optokinetic reflex decreases. When the rabbit is removed from this environment and placed in the dark, eye movements are generated that attempt to restore the adapted stimulus condition without negative feedback. In many rabbits OKAN II would persist at the onset even in an illuminated background since the eyes are moving at velocities that exceed the detection range of direction-selective ganglion cells.

**Figure 11:**
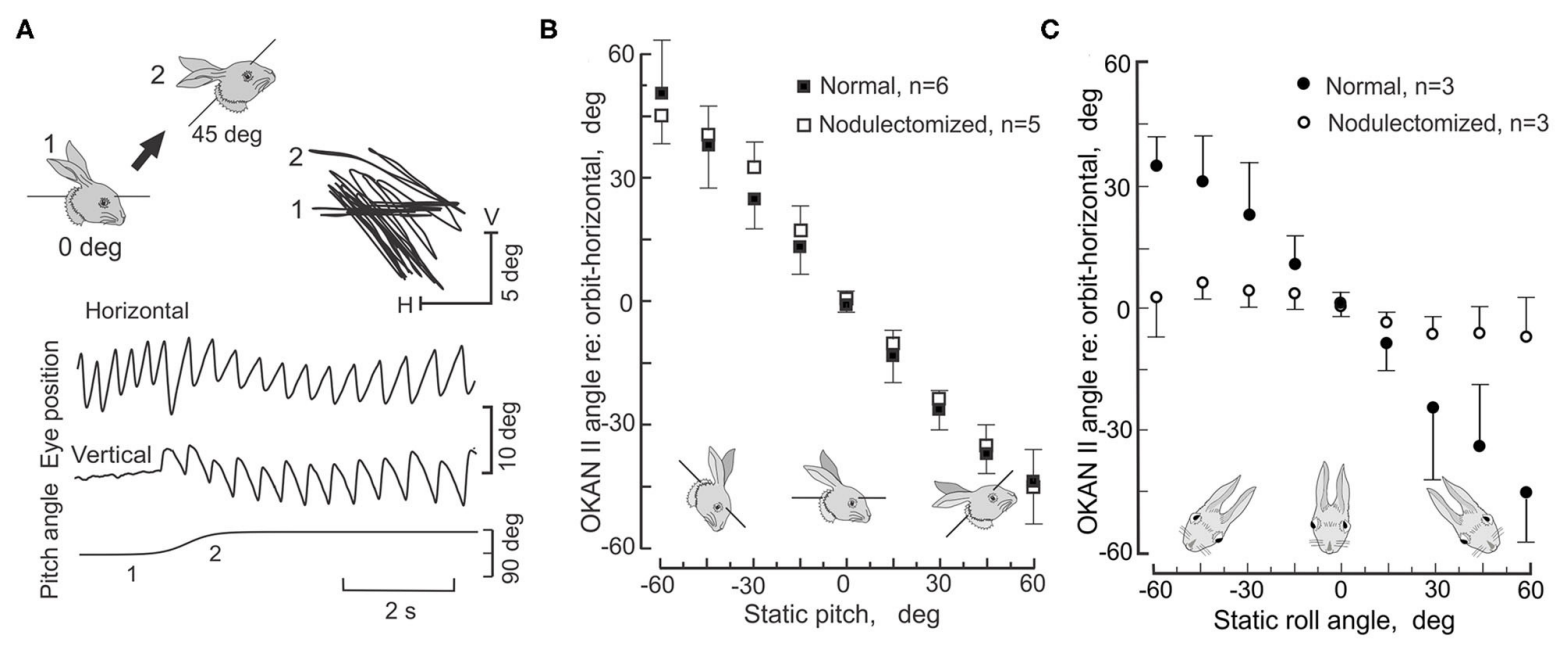
Nodulectomy distorts plane of OKAN II during static roll, but not during static pitch. OKAN II is induced following 24–48 h of binocular HOKS at 5 deg/s. **(A)** During static pitch about the inter-aural axis in normal rabbits, OKAN II remains orthogonal to the gravitational vector (filled circles). This is shown by the sequential change in the vertical and horizontal eye movement records as the rabbit's head position is shifted about the interaural axis by 45 deg as indicated by the figurines. **(B)** OKAN II remains executed in spatial coordinates after a nodulectomy when the rabbit is pitched about the inter-aural axis. **(C)** During roll-tilt about the longitudinal axis, OKAN II adjusts vertically to maintain its horizontal plane in spatial coordinates. After a nodulectomy, OKAN II is no longer executed in spatial coordinates during roll-tilt about the longitudinal axis. Rather it is executed in orbital coordinates. Modified from Barmack et al. (151).

Because OKAN II persists for hours, it is relatively easy to measure the plane of OKAN II and how it is modified by changes when the head is pitched and rolled. In the lateral-eyed rabbit, roll-tilt should yield equivalent results to pitch in monkeys. During rotation about the longitudinal axis in normal rabbits OKAN II remains horizontal in space and compensates for the roll-tilt by moving vertically within the orbit (Figure 11A). OKAN II remains horizontal in space during pitch about the inter-aural axis in both normal and nodulectomized rabbits (Figure 11B). However, in nodulectomized rabbits this gravitational reference is lost (Figure 11C) (151).

Both PRN and OKAN II reflect an imbalance in pre-oculomotor circuitry. It is astounding to consider that during roll-tilt a gravitational vector can be interpreted by cerebellar and brainstem circuitry to perform the same horizontal eye movement in space by executing graded commands for the reciprocal activation of the horizontal and vertical rectus muscles.

During roll-tilt the lobules IX-X are necessary to maintain the plane of OKAN II constant with respect to gravity. This is consistent with the abundance of climbing fiber-encoded signals related to static roll and the absence of climbing fiber-encoded signals from the horizontal canals. Conversely, a nodulectomy has no effect on the gain of the HVOR and causes only a small decrease in the gain of the VVOR. Nor does a nodulectomy disrupt optokinetic suppression of either the HVOR or VVOR (151).

### HOKS Evokes Transcription and Expression of Corticotropin Releasing Factor (CRF)

**Figure 12:**
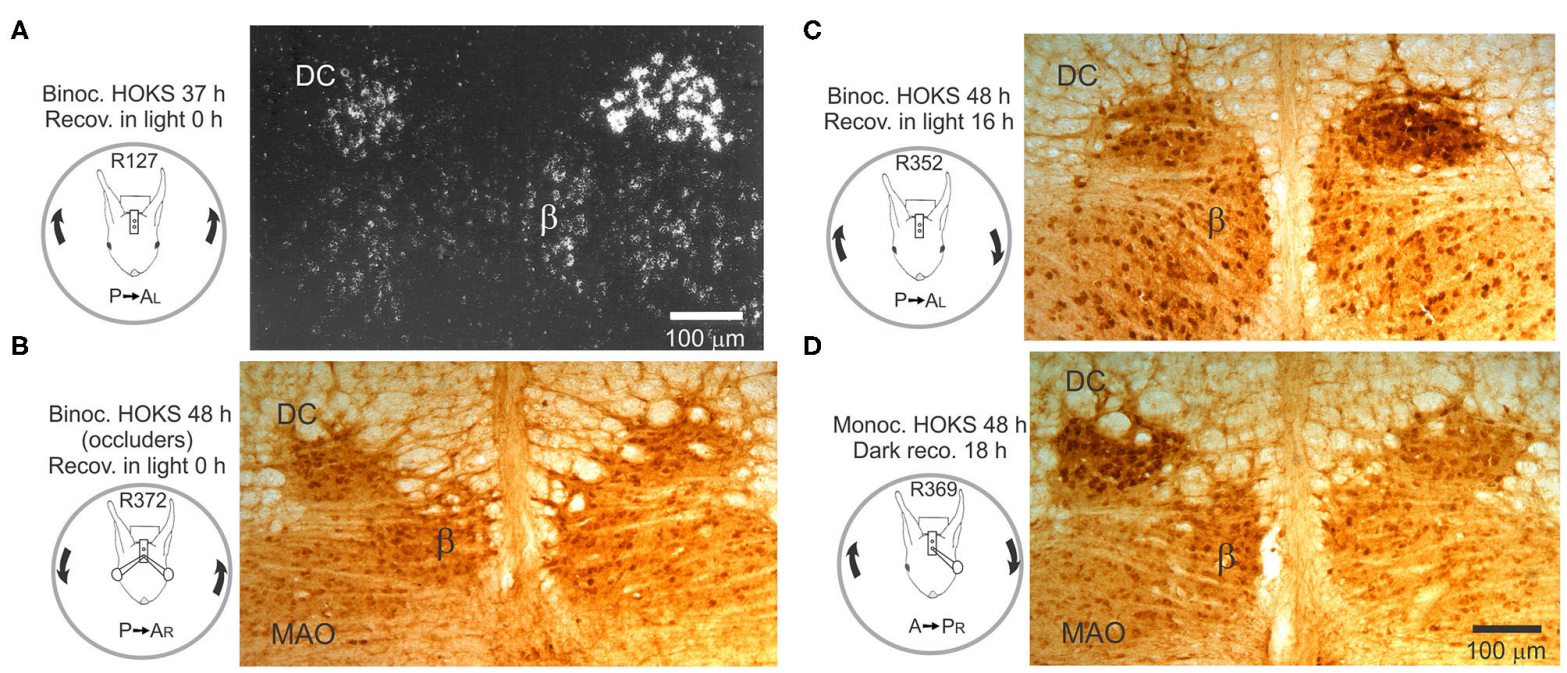
Transcription and expression of corticotropin-releasing factor (CRF) is modified by HOKS. **(A)** Binocular P→A HOKS at 5 deg/s with respect to the right is given for 37 h. The rabbit is then anesthetized and euthanized. Emulsion-coated brainstem sections show increased CRF mRNA over individual neurons in caudal dorsal cap. **(B)** Binocular HOKS is given for 48 h during which vision of both eyes is blocked by ping-pong ball occluders. Brainstem sections are reacted with α-CRF antibody. Neither the left nor right caudal DC has elevated expression of CRF. **(C)** Binocular P→A HOKS is given for 48 h with respect to the right eye, after which the rabbit remains in the dark for 18 h. The right caudal DC has elevated CRF expression. The left caudal DC has a modest increase in CRF expression. **(D)** Monocular A→P HOKS with respect to the right eye is delivered for 48 h while the left eye is occluded. When HOKS stops the rabbit recovers in total darkness for 18 h. During the recovery period the left caudal DC develops increased expression of CRF; an apparent rebound following stimulation in the non-preferred direction. Modified from Barmack (146) and Barmack and Young (152).

Sustained binocular HOKS evokes OKAN II that lasts 24 h or longer, depending on the parameters of stimulation (150). While the cerebellar flocculus has been the focus of experiments designed to test the role of the inferior olivary neurons in visual-vestibular adaptation, changes observed following sustained HOKS suggest that optokinetic adaptation is already present in optokinetic circuitry prior to its entry to the cerebellum (152, 153). Binocular HOKS in rabbits for 16–48 h increases the transcription and expression of corticotropin releasing factor (CRF) in neurons in the caudal DC contralateral to the eye receiving P→A optokinetic stimulation (Figures 12A–D). If the rabbit is anesthetized and euthanized immediately after HOKS is stopped, neurons in the DC contralateral to the eye that received P→A stimulation have elevated transcription of CRF (Figure 12A). Following 24 h of binocular HOKS an estimate of the hybridized grain densities in neurons in the two DCs yield an estimate of a 4-7X increase of CRF mRNA transcripts in neurons in the DC stimulated in the P→A direction relative to the transcripts in neurons in the contralateral DC. If binocular HOKS is given for 48 h and the rabbit is allowed to recover for 16–18 h in the light before it is anesthetized and euthanized, elevated expression of CRF is still found in neurons in the DC contralateral to the eye stimulated in the P→A direction, with a smaller increase in CRF expression in the DC contralateral to the eye stimulated in the AP direction (Figure 12C). If monocular, HOKS in the *null* (A→P) direction is given for 48 h and the animal is allowed to recover for 18 h in the dark, then elevated expression of CRF is found in the DC contralateral to the eye previously stimulated in the AP direction (Figure 12D) (154). HOKS is essential to evoke the change in CRF expression. If contour vision is obscured by translucent occluders then no change in CRF expression in DC neurons on either side of the brain is observed (Figure 12B). After HOKS stops these neurons may evince a rebound excitation when the disfacilitatory signal is removed. OKAN II persists in rabbits that recover in the dark. When rabbits recover in the light, OKAN II is suppressed by visual feedback. This effectively uncouples the retinal slip signal from the eye movement signal. A rebound in activity in the caudal DC previously stimulated in the A→P direction could occur if subsequently the caudal DC receives an OKAN II-associated eye movement signal in the form of reduced inhibition from the contralateral NPH. This explanation is consistent with the idea that floccular CSs encode not just retinal slip, but a mixture of retinal slip and eye movement signals (155).

Cerebellar Purkinje cells, particularly those in hemispheric lobule X and vermal lobules IX-X, express CRF binding sites (156–158). Only one of the two CRF receptor subtypes, CRF_1_, is expressed in the cerebellum (159). CRF receptors belong to the VIP/calcitonin family of G protein-coupled receptors. They are positively coupled to adenylate cyclase. Direct application of CRF onto Purkinje neurons *in vitro* increases excitability attributed activation of a sodium current and a voltage-dependent potassium current (160). In cerebellar cultures, CRF modulates gene expression via a cAMP pathway (161). This pathway could be responsible for the long-term regulation of calcium-activated potassium conductance and thereby account for the decreased after-hyperpolarization observed in Purkinje cells *in vitro* after bath application of CRF (162).

CRF is localized in neurons that comprise the hypothalamo-pituitary-adrenal (HPA) axis, principally engaged in the neuroendocrine response to stress. Sex differences in the HPA axis are manifest in gonadal sex steroids and neuroactive metabolites. CRF expression may be one of the dynamic antecedents of motion sickness.

### HOKS of CSs Evokes Epigenetic Changes in Transcription

Changes in synaptic efficacy observed during LTD last seconds to tens of minutes. The changes in such short-term plasticity involve the translocation of as many as 100 proteins (163). Longer-term changes in synaptic efficacy may also involve changes in gene transcription. Sustained neuronal activity could regulate a variety of transcription factors, each acting on a single gene. Alternatively, neuronal activity could regulate a few transcription factors, each acting on several genes. This second possibility could be realized through the regulation of microRNAs, short, 22 nucleotides, non-proton-coding, RNA that regulates expression of protein-coding mRNAs by repressing their translation or enhancing their degradation (164). Theoretically, a single microRNA may have nucleotide complementarity with as many as 30 different mRNAs, giving it a potential wide regulatory influence (165). Based upon microRNA complementarity, derived from genome databases, as many as one-third of human protein-coding genes may be influenced by microRNAs (166). Functionally, the transcription of most microRNAs is correlated with cellular development, apoptosis (164, 167–172) and microbial defense (164, 173, 174).

### HOKS Evokes Increased Transcription of Several microRNAs

The mouse optokinetic pathway offers the opportunity of learning how repeated activation of a climbing fiber synapse changes transcription of microRNAs in floccular Purkinje cells. Specific microRNAs might suppress mRNAs involved in the expression of target proteins related to cellular adaptation. This is especially attractive because it uses circuitry that is well-described physiologically and known to be critical for adaptation at behavioral and cellular levels. It is also attractive because it allows for the measurement of the subcellular consequences of sustained activation of the most powerful synapse in the central nervous system.

**Figure 13:**
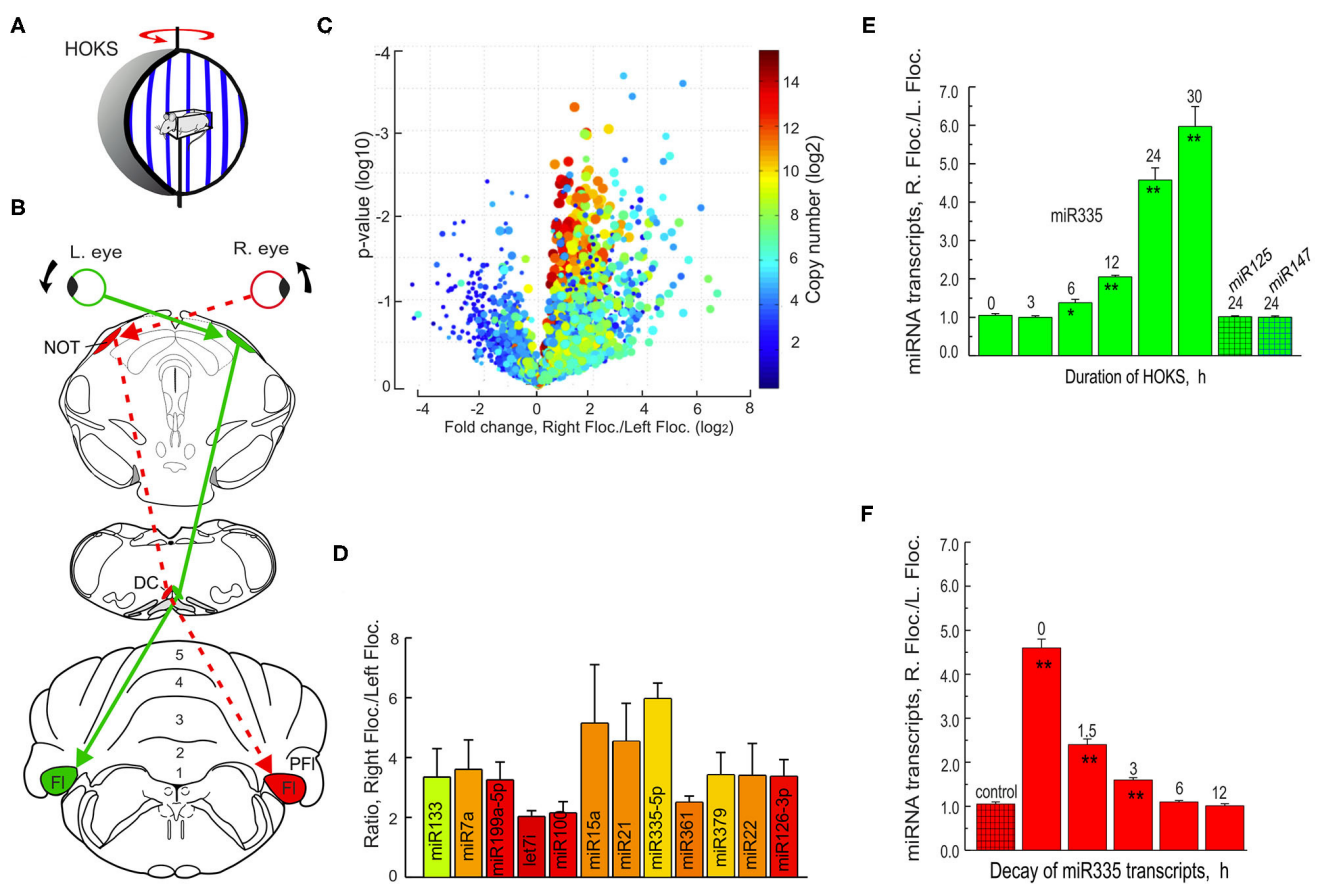
Monocular HOKS increases microRNA transcription in the right flocculus. **(A)** Mice are restrained at the center of an optokinetic sphere. Rotation of the sphere provides P→A HOKS of the right eye at 6 deg/s. The left eye is occluded. **(B)** Functional optokinetic pathway from the right retina to the left DC and right flocculus are depicted. The dashed red lines indicate the excitatory pathway. **(C)** RNA samples from left and right flocculi from the optokinetically stimulated mice are analyzed with a microRNA microarray that includes 616 human and 361 mouse mature microRNAs and are illustrated in a Volcano plot. **(D)** Twelve microRNAs are detected by the microarray in which the ratio of the right flocculus/left flocculus is at least 3X > 1.0. **(E)** The time course of transcription of miR335 is measured following 0-30 h of monocular HOKS. The flocculi are dissected and prepared for RNA extraction. cDNAs are synthesized and amplified by PCR. U6 is co-amplified and used as a loading control. Each paired sample is run on a gel to determine the optical density of the amplified bands. The ratio of PCR band density (RF/LF) shows increased transcription in the right flocculus for miR335. Two control microRNA samples, miR125 or miR147, show no change in transcription. Each histogram bar represents the mean for three mice. **(F)** The decay of HOKS transcripts of miR335 is measured at different intervals following termination of 24 h of HOKS. Statistical significance is indicated by * for *p* < 0.02 and ** for *p* < 0.001. DC, dorsal cap; Fl, flocculus; NOT, nucleus of the optic tract; PFl, paraflocculus. Modified from Barmack et al. (175).

Mice are restrained at the center of an optokinetic sphere rotated CCW at 6 deg/s, stimulating the right eye in the P→A direction and the left eye in the A→P direction. The optokinetic pathway from the right retina to the left DC and then to the right flocculus is indicated by a dashed red line (Figures 13A,B). Tissue samples from both the left and right flocculi are collected, RNA extracted and run on a microRNA microarray. The data set from the microarray are plotted in a Volcano plot that includes information about representation of microRNA transcripts, their copy number, frequency of occurrence, and difference in ratio of transcription of the right floccular sample/left floccular sample (Figure 13C). The microRNAs with the largest differential transcription in the left and right flocculi are identified (Figure 13D).

The single microRNA with the largest differential transcription, miR335, is then PCR-amplified and the ratio of the right/left flocculus measured after HOKS stimulation for different durations. Binocular HOKS for 6 h is the minimal duration of stimulation that induces a detectable increase in transcription of miR335 in the right/left flocculus ratio (RF/LF) (Figure 13E). With increasing duration of HOKS RF/LF increases up to 30 h of HOKS, the experimental limit. At 30 h of HOKS, miR335 RF/LF increased 6-fold relative to unstimulated controls. This optokinetically evoked transcriptional change does not occur in two other microRNAs, miR125, and miR147.

A potential role of microRNAs in any HOKS-induced neuronal adaptation is, in part, dependent on the duration of the RF/LF ratio of transcripts after the stimulus stops. The time course of microRNA decay regulates the duration of suppressive effect on its complementary mRNA target and ultimately on the decreased expression of a protein. By exposing mice to a fixed duration of monocular P→A HOKS (24 h) and then delaying anesthesia and euthanasia for fixed intervals, this question is addressed. HOKS-induced miR335 transcripts decay with a time-constant of ~2.5 h (Figure 13F) (176).

**Figure 14:**
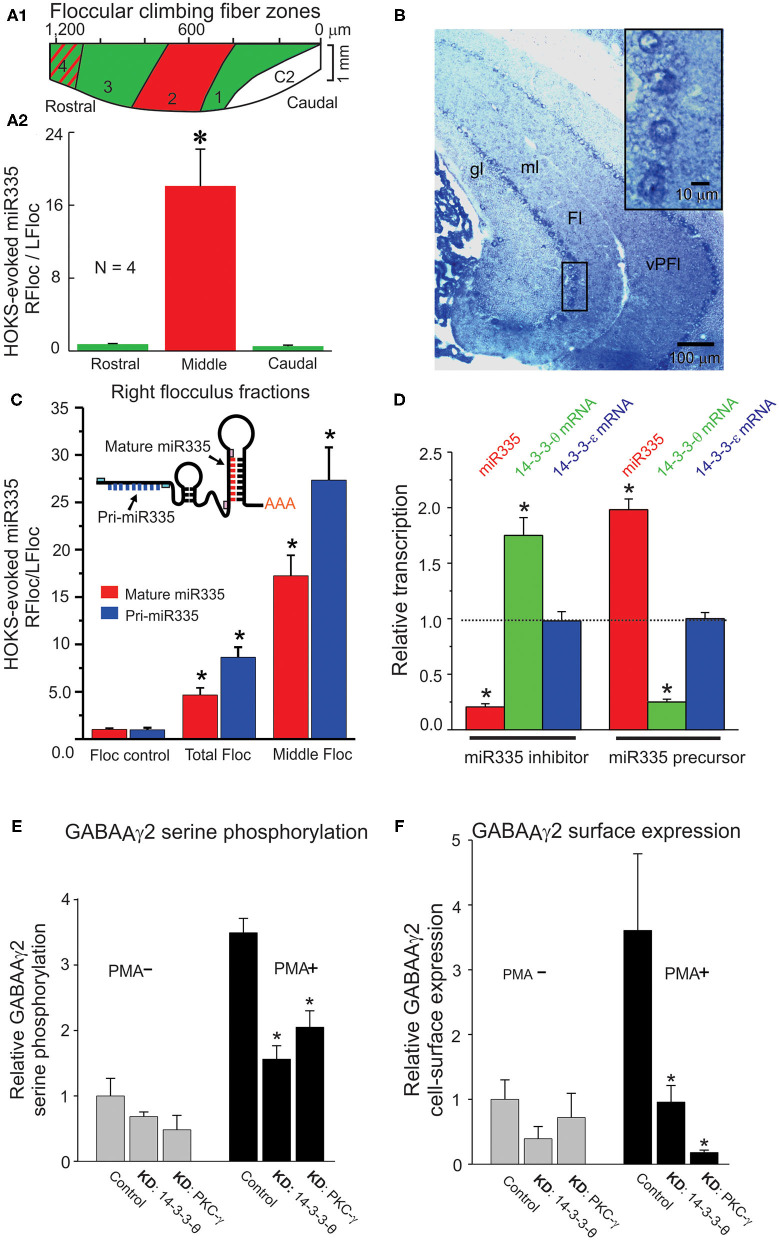
HOKS-evokes transcription of miR335 in Purkinje cells and decreases cell surface expression GABA_A_γ_2_. Three mice receive monocular P→A HOKS of the right eye for 24 h. The right flocculus is divided rostro-caudally, *in situ*, into three fractions from which RNA is extracted separately to obtain a cell fraction (middle) rich in Purkinje cells that respond to HOKS. **(A1)** Planar map of five climbing fiber floccular zones. Purkinje cells in zones 1 and 3 (green) respond to vertical optokinetic stimulation (VOKS). Cells in zone 2 (red) respond to HOKS. Cells in zone 4 (green with red stripes) respond to VOKS and partially to HOKS. Cells in C2 respond to neither HOKS nor VOKS. **(A2)** PCR-amplified cDNAs of miR335 and U6 are shown for each fraction. The ratio of miR335 transcripts in each of the right floccular fractions is plotted relative to transcripts of the unstimulated left flocculus. **(B)** Hybridization histochemistry shows that miR335 is expressed in Purkinje cells. Transverse sections through the cerebellar flocculus and paraflocculus are hybridized to a digoxigenin-labeled oligonucleotide complementary to miR335 and immunolabeled with an antibody to digoxigenin. Four Purkinje cells, denoted by a boxed outline, are shown at higher magnification in upper right corner. The oligonucleotide probe immuno-labels cytoplasm surrounding unlabeled nuclei. **(C)** Both intranuclear pri-microRNA and mature microRNA transcripts are measured to examine whether changes in mature microRNA accurately reflect changes in transcription. qPCR is used to identify miR335 both pri-miR335 and mature miR335 using specific primer pairs as shown in the cartoon. Following 24 h of monocular P→A HOKS of the right eye both pri-miR335 and mature miR335 transcripts increase. **(D)** miR335 reduces transcripts of 14-3-3-θ *in vitro*. Treatment of N2a cells with the miR335 inhibitor decreases miR335 transcripts, increases 14-3-3-θ transcripts, and has no effect on 14-3-3-θ transcripts. Treatment with the miR335 precursor increases miR335 transcripts, decreases 14-3-3-θ transcripts, and has no effect on 14-3-3-θ transcripts. In N2a cells, two native proteins, 14-3-3-θ and PKC-γ are knocked down independently by siRNA treatment or miRNA transfection. The N2a cells are “stimulated” by treatment with 200 nM of phorbol 12-myristate-13-acetate (PMA), a PKC activator, or they are “not stimulated” with no PMA treatment. **(E)** PMA treatment increases serine phosphorylation of GABA_A_γ_2_ in N2a cells. Knockdown of 14-3-3-θ (KD: 14-3-3-θ or PKC-γ (KD: PKC-γ decreases serine phosphorylation only in cells treated with PMA. **(F)** Knockdown of 14-3-3-θ or PKC-γ decreases cell surface expression of GABA_A_γ_2_ in N2a cells “stimulated” by PMA. Cell-surface expression of GABA_A_γ_2_ is measured by selective biotinylation of membrane proteins. (ANOVA, *p* < 0.001 indicated by asterisk). Fl, flocculus; gl, granule cell layer; ml, molecular layer; vPFl, ventral paraflocculus. Modified from Schonewille et al. (92), Barmack et al. (176), Barmack et al. (175), and Qian et al. (177).

Following HOKS, microRNAs can be measured with greater accuracy by collecting floccular samples from the zone within the flocculus that contains only Purkinje cells whose discharge is modulated by HOKS. This is possible because the topography of optokinetically responsive floccular Purkinje cells in the mouse has already been obtained (92) (Figure 14A1). The spindle-shaped single folium flocculus is ~1.2 mm long and includes five separate regions classified by their sensitivity to optokinetic stimulation about three axes. Purkinje cells in the central region respond to HOKS. This central region is flanked by Purkinje cells that respond to stimulation about the anterior and posterior axes (92) (Figure 14A2). Using these improved floccular samples, the right “stimulated” miR335 transcripts increase 18X relative to the “unstimulated” left flocculus miR335 transcripts after 24 h of HOKS.

Increased miR335 transcripts in a cerebellar lysate is not proof that the increase can be localized to Purkinje cells. However, hybridization histochemistry localizes miR335 transcripts to Purkinje cells (Figure 14B). The inserted box shows at a higher magnification that the miR335 hybridization probe labels Purkinje cell cytoplasm, not the nucleus.

The increase and subsequent decay of microRNA transcripts in the flocculus after HOKS could indicate differences in miR335 transcription or changes in cytoplasmic post-transcriptional events or both. Nuclear transcription of microRNAs is preceded by transcription of larger pri-microRNAs. A variety of enzymatic post-transcriptional factors could contribute to the regulation of microRNAs. These factors might confound interpretations concerning increases or decreases in cytoplasmic microRNA transcripts and whether such changes can be attributed to transcription or to post-transcriptional cytoplasmic regulatory events. This question can be answered by measuring transcripts of pri-miR335 and mature miR335 in floccular mRNA samples extracted from “stimulated” and “non-stimulated” flocculi (Figure 14C). Following 24 h of HOKS, pri-miR335 transcripts from the middle zone of the stimulated (right) flocculus increase 28X relative to the transcripts in the unstimulated (left) flocculus. Mature miR335 transcripts increase by 18X.

The nucleotide sequence of miR335, analyzed with the use of two data bases offers a long list of clues as to the likely complementary mRNA targets. However, even under the most stringent conditions, these lists are unacceptably large. The microRNA Registry and EnsEMBL propose more than 149 mRNAs with sufficient complementarity to hybridize with miR335.

As an alternative, mRNA arrays can screen mRNAs whose transcripts decrease after 24h of HOKS. A total of 42 such mRNA transcripts are reduced in the right flocculus by P→A HOKS of the right eye for 24 h (176, 178). Using a more direct approach, miR335 inhibitors can be transfected directly into the posterior vermis to assess possible changes in mRNA transcripts. Combining results from these three methods: [1] miR335 is predicted to be complementary to many transcripts including transcripts of calbindin and 14-3-3-θ a regulatory protein that binds to functionally diverse signaling proteins (179, 180), [2] The transcripts of many proteins including calbindin and 14-3-3-θ decrease following 24 h of HOKS, and [3] Transcripts of calbindin and 14-3-3-θ increase after injection of miR335 inhibitors into cerebellar tissue (175).

### Interactions of microRNA and mRNA Transcripts Are Examined in N2A Cells

The interaction of miR335 with its complementary targets could be achieved through interactions within the cerebellar cortex also induced by climbing fiber activity. Alternatively, interactions of miR335 with its target mRNAs can be examined directly *in vitro*. N2a cells serve as a model system for detecting interactions of miR335 with calbindin and 14-3-3-θ. These three components are expressed natively in N2a cells. The effects of transfecting N2a can with miR335 inhibitors or miR335 precursors on mRNA transcripts for miR335, 14-3-3-θ calbindin and 14-3-3-θ (control) can be measured directly using quantitative PCR. When N2a cells are transfected with a miR335 inhibitor, transcripts of miR335 decrease and transcripts of 14-3-3-θ increase. Transfection of N2a cells with a miR335 precursor increases transcripts of miR335 and decreases transcripts of 14-3-3-θ. The specificity of these effects is strengthened by the lack of changes in 14-3-3-θ transcripts, a control isoform (Figure 14D).

### 14-3-3-θ, PKC-γ, and GABA_A_γ_2_ Are Functionally Linked in Purkinje Cell GABA_A_ Receptors

Increased transcription of miR335, evoked by climbing fiber excitation of Purkinje cells, decreases transcripts of 14-3-3-θ. In cerebellar lysates 14-3-3-θ interacts with PKC-γ, one of numerous constitutively-expressed isoforms of protein kinase C. PKC-γ phosphorylates several proteins expressed in the cerebellum. Immunoprecipitation of 14-3-3-θ co-immunoprecipitates PKC-γ. Immunoprecipitation of 14-3-3-θ also co-immunoprecipitates GABA_A_γ_2_, a subunit of the pentameric GABA_A_ receptor involved with its membrane assembly (177, 181–186). This role of these proteins in the assembly of GABA_A_ receptors can be examined in N2a cells in which these proteins are native as is the GABA_A_ receptor. To mimic synaptic activation of N2a cells they are “stimulated” by treatment with 200 nM of phorbol 12-myristate-13-acetate (PMA), a PKC activator. “Stimulated” cells can be compared with “unstimulated” control cells not treated with PMA. 14-3-3-θ and PKC-γ are “knocked down” by transfecting N2a cells with siRNAs or miRNAs designed specifically to knockdown either 14-3-3-θ or PKC-γ. The objective is to examine how 14-3-3-θ and PKC-γ contribute to the serine phosphorylation of GABA_A_γ_2_ in two groups of N2a cells, one treated with 200 nM of PMA and one untreated control. The outcome of this test is that knockdown of either 14-3-3-θ or PKC-γ reduces serine phosphorylation of GABA_A_γ_2_ only in N2a cells that also are activated by PMA (Figure 14E).

The possibility that knockdown of either 14-3-3-θ or PKC-γ reduces the cell surface expression of GABA_A_γ_2_ is examined with the use of an assay that preferentially biotinylates cell surface proteins (177). Again, one can compare the efficacy of the knockdowns in N2a cells that are treated with PMA in contrast to those that are untreated (Figure 14F). In PMA treated N2a cells knockdowns of either 14-3-3-θ or PKC-γ reduce cell surface expression of GABA_A_γ_2_.

If the same interactions of microRNA that occur in N2a cells also occur in Purkinje cells then a possible homeostatic mechanism is at least one of the consequences of sustained HOKS as follows: The gain of the horizontal optokinetic reflex slowly attenuates with sustained HOKS. Sustained HOKS increases transcription of miR335. Increased miR335 transcripts block expression of 14-3-3-θ and PKC-γ mRNAs thereby reducing the serine phosphorylation of GABA_A_γ_2_ and reducing its insertion into the post-synaptic membrane. Fewer GABA_A_ receptors reduces inhibition of Purkinje cells and perhaps maintain a constant level of excitability during sustained stimulation.

### Plasticity of Neurons in the Medial Vestibular Nucleus

**Figure 15:**
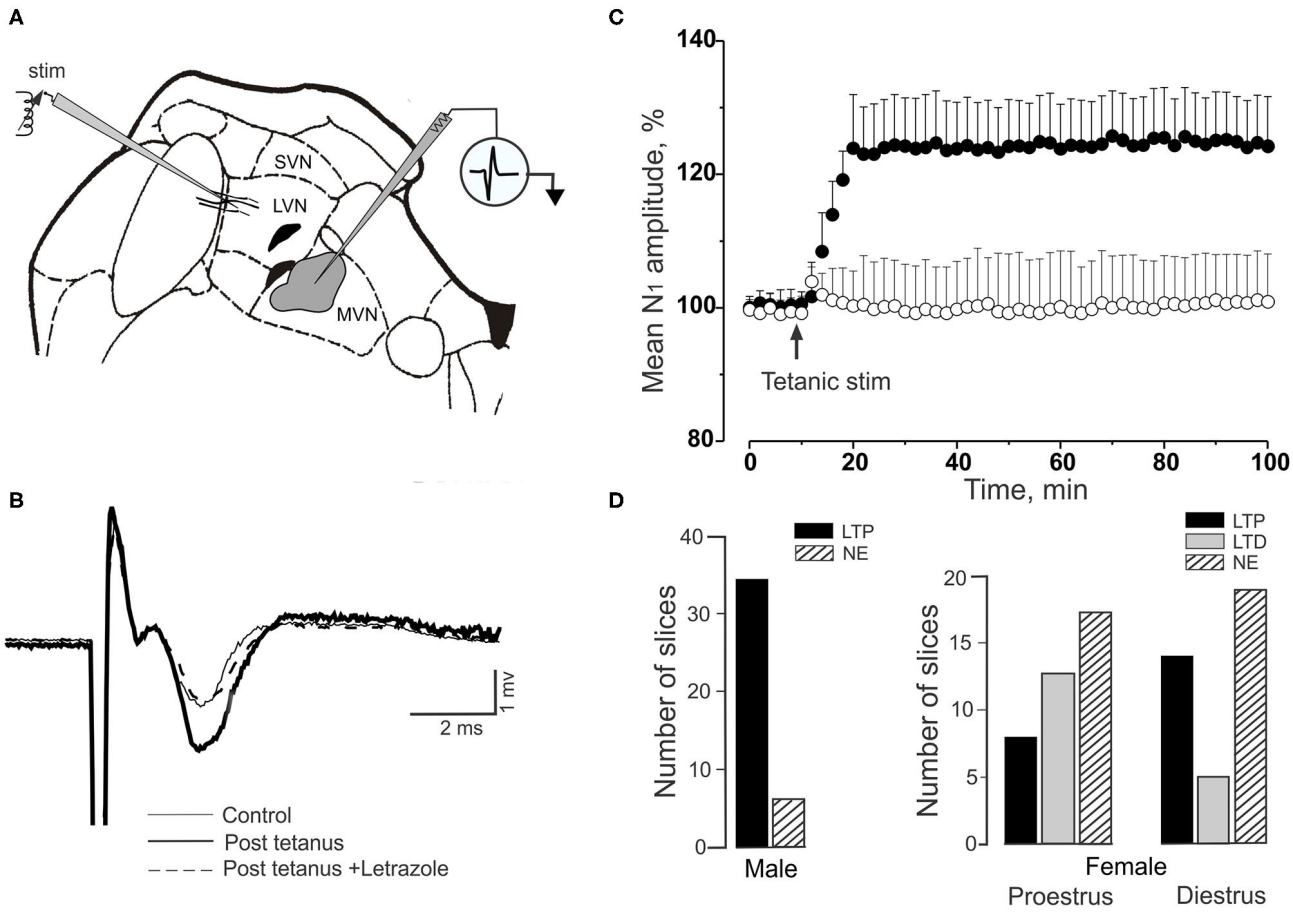
Induction of long-term potentiation (LTP) in medial vestibular nucleus (MVN) neurons differs in brain slices of male and female rats. **(A)** Tetanic stimulation (Four trains, 1 s and 100 Hz, are applied to the vestibular afferents distal to their termination in the MVN. The zone of recording is demarcated by gray shading. **(B)** Field potentials from the MVN evoked by single shock stimulation to vestibular afferents in rat brain stem are illustrated. The thin trace is evoked prior to tetanic stimulation. The thicker trace is recorded after tetanic stimulation. The dashed trace is evoked after blockade of neurosteroid 17beta-Estradiol (E2) synthesis by Letrozole. **(C)** Time course of field potential amplitude in two male rats. In one slice LTP is induced by tetanic stimulation (filled circles). In the other LTP is prevented with bath-applied aromatase inhibitor, Letrozole (open circles). In both conditions the symbols indicate means and SDs of 8 responses to stimuli delivered every 15 s. **(D)** The histograms compare long-term effects induced by tetanic stimulation in male, proestrus and diestrus of female rats. Left histogram panel shows number of experiments with LTP in male brain slices (black bar) and the number of male brain slices in which tetanic stimulation fails to evoke LTP (NE, striped bar). Right panels show female brain slices tested during proestrus and diestrus. In the brain slices of females, LTP (black bars) is reduced in frequency, compared with LTP in male slices. In both proestrus and diestrus females LTD is induced (gray bars), while LTD is not induced in male slices. In proestrus, the incidence of LTP is lower than that in diestrus, suggesting that the level of the circulating E2 interferes with local neurosteroid E2 synthesis required for LTP induction. LVN, MVN, SVN, lateral, medial and superior vestibular nuclei. Modified from Grassi et al. (191).

Secondary vestibular neurons share with cerebellar Purkinje cells adaptive characteristics that may account for vestibular compensation and visuo-vestibular calibration (187–190). In secondary vestibular neurons this property depends on changes in the cellular excitability and synaptic responsiveness (191, 192). This adaptation can be investigated in brainstem slices in which high frequency tetanic stimulation of vestibular primary afferents evokes long-term potentiation (LTP) in secondary neurons of the medial vestibular nucleus (MVN). LTP is evoked through the activation of metabotropic glutamate type-1 NMDA receptors. On the other hand, low frequency stimulation evokes long-term depression (LTD) (193). The more relevant characteristic of the stimulation for stimulus-induced plastic changes in EPSP amplitude is the temporal organization of repetitive bursts, probably causing different intracellular levels of postsynaptic Ca2+. Short burst intervals (four trains, 1 s, 100 Hz) induce LTP, longer burst intervals induce LTD. Temporal stimulation sequencies may assure a persistent selectivity of the MVN neuron in response to input from semicircular canals, otolithic receptors and optokinetic signals or to cerebellar output. Moreover, the induction of vestibular LTP and LTD is influenced by sex neurosteroids, as occurs in several areas of the CNS (194). In the MVN 17 beta-Estradiol (E2**)** and 5α-dihydrotestosterone (DHT) rapidly and oppositely regulate synaptic transmission and plasticity by directly interacting with membrane estrogen and androgen receptors (ERs and ARs) (195–197). Estradiol mediates the LTP, while DHT mediates LTD (Figures 15A–C). A similar influence of estradiol is found in cerebellar Purkinje neurons (198, 199).

Both the occurrence and amplitude of LTP in the MVN of female rats is altered at different phases of the cycle (192, 200). Specifically, high frequency stimulation induces LTP in male rats within seconds (fast-developing LTP). However, this same stimulation elicits variable long-term synaptic effects in female rats; fast-developing LTP or slow-developing LTP or even LTD. LTP amplitude and frequency of occurrence depends on the neural E2 levels, fluctuating according to oestrous phases, with a high probability of inducing fast LTP in dioestrus phase (Figure 15D). A high E2 level decreases LTP by preventing the transformation of testosterone into E2 and facilitating its transformation into DHT (192).

### Cerebellar Functions and Cellular Mechanisms

The zonal architecture of visual and vestibular climbing fiber projections to hemispheric and vermal lobules IX-X establishes a three-dimensional optokinetic and vestibular space. These sagittal zones are defined by their afferents and by their projections. The climbing fiber afferents appear to be the primary regulators of Purkinje cell SS discharge frequency. In vermal lobule X, vestibular primary afferent→granule cell→parallel fiber→Purkinje cell signals originate from the three ipsilateral semicircular canals and two otolith organs. These primary afferent signals are mixed with secondary afferent signals from the vestibular nuclei. This mixture of signals modulates the discharges of Purkinje cells in the medial sagittal zone by climbing fiber signals that originate from the contralateral anterior semicircular canal and utricle. More rostrally, in vermal lobules IXa,b, primary vestibular afferents are absent. In their absence, secondary vestibular afferents remain and perhaps additional mossy fiber afferents from neck and shoulder muscle proprioceptors are added to the mix. Climbing fiber projections that originate from the contralateral anterior semicircular canal and utricle persist. In both the caudal and rostral zones, the modulated polarity of CSs and SSs remains the same, although the depth of modulation may vary depending on the intensity of parallel fiber discharge. The outputs of sagittal climbing fiber zones are distributed along a longitudinal gradient to the cerebellar nuclei, vestibular nuclei and other brainstem nuclei. The functions of these sagittal zones encompass a more global view of the same basic signal tailored to different functional subnuclei all of which require an accurate reading of head orientation in space.

Spontaneous increases or decreases in SS discharge can, on occasion, precede as well as follow the occurrence of CSs in the same Purkinje cell (64, 201–204). Under certain stimulus and recording conditions the modulation SS discharge occurs in the absence of modulation of CS discharge. In these instances, the SSs are often regarded as a consequence of a mossy fiber→granule cell→parallel fiber→Purkinje cell throughput that is independent of the climbing fiber pathway with the possible exception of undefined “novel” responses evoked by climbing fibers (201–204). Given the significance of sagittal zones in lobules IX-X it would seem useful to learn if zonal architecture also influences the responses of Purkinje cells in other cerebellar lobules. In lobules IX-X it has been helpful to deploy a variety of well-controlled vestibular and optokinetic stimuli to investigate the discharge characteristics of Purkinje cells. Perhaps a refinement of stimulus conditions would similarly be useful for investigating other cerebellar regions.

At a more molecular level, the potential role of NMDA receptors in homeostatic regulation of the discharge of MVN neurons (205) and cerebellar stellate cells (139, 140) offers the possibility of enhancing our understanding of cellular plasticity within a behavioral context.

Epigenetic factors also may contribute to dynamic regulation of climbing fiber discharge. Stimulus-evoked changes in microRNA transcription provides one of several ways to investigate epigenetic factors that may regulate the synaptic efficacy of Purkinje cell discharge. Climbing fiber-evoked increases in microRNA transcription in Purkinje cells suggests that microRNAs contribute to the homeostatic regulation of proteins critical for Purkinje cell synaptic function. The trick will be to learn how the suppressive effects of microRNAs contribute to the regulation of proteins critical for maintaining and altering synaptic function.

## Author's Note

Cerebellar adaptation to vestibular and optokinetic depends on specialized circuitry that represents three dimensions in a space in which the coordinates correspond physically to the orientation of the semicircular canals and utricular otolith. This circuitry is localized to the hemispheric and vermal lobule X (flocculus and nodulus). The nodular coordinate system senses gravity and head movement around the axes of the vertical semicircular canals and utricular otolith. The flocculus senses low velocity optokinetic stimulation about three axes in the absence of a gravity vector. We review three aspects of vestibular and optokinetic adaptation in which the flocculus and nodulus play an important role. Cerebellar adaptation to sustained optokinetic or vestibular stimulation is revealed physiologically in the persistence of responses after initial stimulation has ceased. It is also shown behaviorally in the persistence of eye movements in the absence of the stimulation that first evoked them. We link these sensory adaptations to epigenetic signaling in Purkinje cells and to stimulus-evoked expression of sex-related peptides, corticotropin releasing factor and neurosteroid 17beta-Estradiol (E2). We suggest that these peptides may also explain, in part, the female predominance in the mal de debarquement disorder.

## Author Contributions

All authors listed have made a substantial, direct and intellectual contribution to the work, and approved it for publication.

## Conflict of Interest

The authors declare that the research was conducted in the absence of any commercial or financial relationships that could be construed as a potential conflict of interest.
